# Cytochrome P450 1A1 enhances inflammatory responses and impedes phagocytosis of bacteria in macrophages during sepsis

**DOI:** 10.1186/s12964-020-0523-3

**Published:** 2020-05-04

**Authors:** Li-Xing Tian, Xin Tang, Jun-Yu Zhu, Li Luo, Xiao-Yuan Ma, Shao-Wen Cheng, Wei Zhang, Wan-Qi Tang, Wei Ma, Xue Yang, Chuan-Zhu Lv, Hua-Ping Liang

**Affiliations:** 1 State Key Laboratory of Trauma, Burns and Combined Injury, Department of Wound Infection and Drug, Daping Hospital, Army Medical University, Yuzhong District, Chongqing, China; 2 Trauma Center, The First Affiliated Hospital of Hainan Medical University, Haikou, China; 3 Emergency and Trauma College of Hainan Medical University, Haikou, China

**Keywords:** Cytochrome P450 1A1, Inflammation, Phagocytosis, Macrophages, Sepsis

## Abstract

**Abstract:**

The hydroxylase cytochrome P450 1A1 (CYP1A1) is regulated by the inflammation-limiting aryl hydrocarbon receptor (AhR), but CYP1A1 immune functions remain unclear. We observed CYP1A1 overexpression in peritoneal macrophages (PMs) isolated from mice following LPS or heat-killed *Escherichia. coli* (*E. coli*) challenge. CYP1A1 overexpression augmented TNF-α and IL-6 production in RAW264.7 cells (RAW) by enhancing JNK/AP-1 signalling. CYP1A1 overexpression also promoted 12S-hydroxy-5Z,8Z,10E,14Z-eicosatetraenoic acid (12(S)-HETE) production in activated RAW, while a 12(S)-HETE antibody attenuated and 12(S)-HETE alone induced inflammatory responses. Macrophages harbouring hydroxylase-deficient CYP1A1 demonstrated reduced 12(S)-HETE generation and LPS-induced TNF-α/IL-6 secretion. CYP1A1 overexpression also impaired phagocytosis of bacteria via decreasing the expression of scavenger receptor A (SR-A) in PMs. Mice injected with CYP1A1-overexpressing PMs were more susceptible to CLP- or *E. coli*-induced mortality and bacteria invading, while Rhapontigenin, a selective CYP1A1 inhibitor, improved survival and bacteria clearance of mice in sepsis. CYP1A1 and 12(S)-HETE were also elevated in monocytes and plasma of septic patients and positively correlated with SOFA scores. Macrophage CYP1A1 disruption could be a promising strategy for treating sepsis.

Video abstract

**Graphical abstract:**

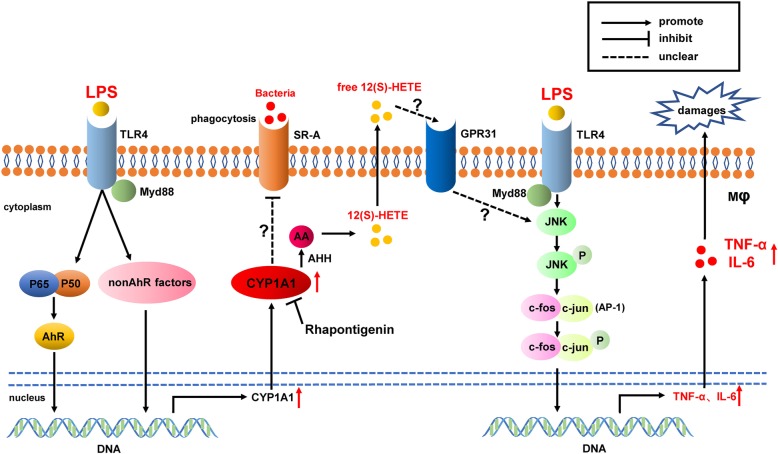

## Background

Sepsis is one of main causes of death in the intensive care unit. It occurs when the immune system becomes dysfunctional in response to existing infection and triggers a cascade of inflammatory responses [1]. Although there are a number of pharmacological interventions for curing sepsis, mortality remains unacceptably high [2]. Thus, promising targets and effective pharmacological treatments for septic patients are urgently needed. Emerging evidence suggests that inflammatory factors such as TNF-α and IL-6 released by hyperactivated macrophages enhance the damaging responses during sepsis [3]. The Gram-negative bacterial LPS generally plays a beneficial role in controlling bacterial infections, but it is also a major driver of aberrant inflammatory factors production in lethal sepsis via binding to Toll Like Receptor 4 [4–6]. In addition, ingestion of bacteria in macrophages has been well-documented as a critical tache in host defence against pathogen invading [7]. Thus, it is essential to elucidate the fundamental mechanisms underlying LPS- and bacteria-induced macrophage overactivation and phagocytosis during sepsis.

CYP1A1 is involved in the metabolism of a broad spectrum of xenobiotics [8–10]. However, several investigations have reported that CYP1A1 also play critical roles in oxidative stress injury, as well as in *mycoplasma* and *Citrobacter rodentium* infection [11–17]. It has been reported that CYP1A1 is a critical enzyme mediating the metabolism of arachidonic acid (AA) to 12(S)-HETE through its hydroxylase activity [18–20]. AP-1, a transcriptional factor composed of c-jun and c-fos families, is a well-documented regulator of inflammatory responses by LPS-induced macrophages [21] and can also be activated by 12(S)-HETE [22–24]. It has been reported that TCDD, an AhR-related inducer of CYP1A1, enhances the DNA binding activity of AP-1 in normal Hepa cells, but not cells expressing hydroxylase-deficient CYP1A1 [25], suggesting a potential relationship between CYP1A1 and AP-1. However, no studies so far have investigated the relationships among CYP1A1, 12(S)-HETE and JNK/AP-1 in macrophages during inflammation or sepsis.

In this study, we identified CYP1A1 as a critical regulator of inflammatory responses and phagocytosis in sepsis and described two novel CYP1A1-invovled signalling pathways, CYP1A1–12(S)HETE−JNK − AP-1 and CYP1A1-SR-A, that may be promising targets for treating sepsis or other inflammatory diseases.

## Methods and materials

### Materials

LPS (*Escherichia coli* 0111: B4) and PMA was purchased from Sigma-Aldrich. 12(S)-HETE from Cayman, and 12(S)-HETE-blocking antibody from ENZO 12(S)-HETE ELISA kit, JNK inhibitor SP600125, AP-1 inhibitor PNRI-299, 12-LOX inhibitor ML355 were produced by MedChemExpress. CYP1A1 inhibitor Rhapontigenin were produced by Santacruz. SR-A monoclonal antibody was purchased from Serotec. Penicillin, streptomycin, puromycin, RPMI 1640 and foetal bovine serum (FBS) were obtained from Gibco-BRL Invitrogen. Ficoll Paque PLUS was purchased from GE Healthcare Life Sciences.

### Preparation of *E.coli* cells

*E.coli* cells (25922, ATCC) were seeded on LB agar plates and cultured at 37°C for commonly maintaining in our lab. One colony from these growing LB agar plates were transplanted into 100 ml of fresh sterile LB medium and incubated on a orbital shaker at 37 °C for 12 h and then transferred to 500 ml of fresh sterile LB medium for another 12 h. The viable *E.coli* cells were harvested by centrifugation at 10000 × g for 5 min and washed by 0.9% NaCl sterile solution, and then resuspended by sterile glycerine. The *E.coli* cells were incubated in a water bath at 90 °C for 15 min for inactivation (heat kill).

### Mice

Healthy C57BL/6 mice (male, 10−12 weeks, 20−25 g) were provided by the Experimental Animal Center of Army Medical University (Chongqing, China). AhR^+/-^ mice, inbred C57BL/6 back-ground, were born and raised in indoor barrier maintained animal facilities at The Jackson Laboratory. WT and AhR^-/-^ were bred from AhR^+/-^ mice and raised in isolation with Specific Pathogen Free status. All experimental procedures and animal welfare protocols were conducted in accordance with the guidelines for laboratory animal care of the National Institutes of Health and Army Medical University.

### *In vitro* culture

#### Mice peritoneal macrophages

Healthy C57BL/6 mice were intraperitoneal injected with 4% thioglycolate for cell extraction. After 3 days’ stimulation, macrophages were extracted from mice by douching the peritoneal cavity with 5 ml cold phosphate buffer saline (PBS). Total extracted cells were centrifuged for 5 min at 300 g and seeded onto Petri dishes for 3 h at 37 °C. Non-adherent cells were removed by washing with PBS, and the adherent cells were harvested for future experiments.

#### RAW264.7 cell line

RAW264.7 cells (ATCC) were cultured in PRMI 1640 medium supplemented with 100 mg/ml streptomycin, 100 U/ml penicillin and 10% FBS at 37 °C under a 5% CO_2_/ sterile air atmosphere. RAW264.7 cells were stably transfected with six types of recombinant lentivirus (GeneChem): 1. Lentivirus containing the whole coding sequences of CYP1A1 and enhanced (E)GFP. 2. Lentivirus containing the sequences of a hydroxylase-deficient CYP1A1 mutant and EGFP. 3. Lentivirus encoding a JNK CRISPR/CAS9 knockout system. 4. Lentivirus encoding a c-fos/c-jun CRISPR/CAS9 knockout system. 5. Lentivirus encoding a JNK CRISPR/CAS9 knockout system and CYP1A1−EGFP. 6. Lentivirus encoding a c-fos/c-jun CRISPR/CAS9 knockout system and CYP1A1−EGFP. The CYP1A1 hydroxylase mutant form was based on the c37 mutant used in previous study [26]. Successfully transfected cell lines were selected and maintained in the presence of 4 *μ*g/ml puromycin.

#### Isolation of primary peripheral monocytes

Fresh blood was collected from consenting healthy and septic donors according to an institutional review board protocol approved by the ethics committee of Army Medical University or *E.coli*-treated mice, respectively. Whole blood was diluted 1:2 in PBS, layered over the lymphosep density separation medium Ficoll Paque PLUS, and concentrated by centrifugation for 20 min at 200 g. Mononuclear cells were washed 3 times with HEPES to remove platelets, isolated using a negative depletion kit (Invitrogen), and then maintain at 37 °C under a 5% CO_2_/ sterile air atmosphere. Some isolated human peripheral monocytes were coaxed into macrophages by PMA (100 ng/ml) for 24 h for indicated experiments.

#### Quantitative reverse transcription PCR

The total RNA was isolated from treated cells using TRIzol reagent (Invitrogen). 1 g cDNA was synthesized from quantified RNA according to the manufacturer’s recommendations (TaKaRa) and the target mRNAs were quantified by qRT-PCR using SYBR Premix (TaKaRa) on a BioRad CFX96. All primer sequences used in this study are listed in supplemental table 1.

#### ELISA

The concentrations of TNF-α and IL-6 in the cell supernatants, PLF and plasma were determined by ELISA kits (Boster) according to the manufacturer’s instructions (450 nm absorbance). The levels of 12(S)-HETE in above samples were measured using a 12(S)-HETE ELISA kit (Cayman) at 405 nm absorbance. The level of 14, 15-EET was assessed using ELISA kit (Detroit R&D) following the manufacture’s instruction.

#### Aspartate Transaminase (AST) and Alanine Transaminase (ALT) Measurements

The blood samples were separated by centrifugation (300 g, 10 min) and the serum portion was sent to the Department of Laboratory Medicine, Daping Hospital, Army Medical University, for quantification of AST and ALT.

#### Western blotting

Treated cells were lysed on ice for 30 min and the supernatant cleared by centrifugation at 12000 g for 15 min at 4 °C. Equal amounts of protein per gel lane were separated by 10% SDS-PAGE and transferred to PVDF membranes. Membranes were blocked with 5% skim milk for 1 h in Tris-buffered saline containing 0.1% Tween 20 (TBST) at room temperature (RT), and then incubated overnight at 4 °C in primary antibodies. The blotted membranes were washed 3 times with TBST and probed for 1 h with appropriate HRP-conjugated secondary antibodies (Cell Signalling Technology). The immunoreactive bands were visualized using an enhanced chemiluminescence detection system (Bio-Rad). The relative intensities of bands were measured by ImageJ software. CYP1A1, 12-LOX, phosphorylated-p50, p50, phosphorylated-JNK and total JNK antibodies were purchased from Abcam. Antibodies against phosphorylated-c-jun, phosphorylated-c-fos, phosphorylated-p65, phosphorylated-ERK_1/2_, phosphorylated-p38, c-jun, c-fos, p65, ERK_1/2_, p38, GPADH and β-actin were purchased from Cell Signalling Technology*.* All antibodies mentioned above are diluted 1:1000 for western blot analysis.

#### Target protein knockdown by RNA interference

CYP1A1 and scrambled control siRNA were provided by GenePharma. Macrophages were seeded at 2 × 10^6^ cells per well (CYP1A1/RAW cells were seeded at 5 × 10^5^ cells per well) on six-well plates and incubated with 120 nM scramble siRNA or target siRNA in Lipo2000 Reagent (GenePharma) for 8 h. The transfection medium was removed and replaced by fresh RPMI 1640 containing 12% serum. After 48 h transfection, cells were then stimulated with 12(S)-HETE or LPS as indicated times.

#### Electrophoretic mobility shift assay

Nuclear proteins were extracted from treated cells using nuclear and cytoplasmic protein extraction kits (Beyotime) following standard procedures. Binding of nuclear extracts to AP-1-binding sites was assessed by electrophoresis of equal amounts of protein and DNA-protein complex on 6% polyacrylamide native gels for 50 min at 160 V. Then the gels were detected at 700 nm using an Odyssey scanning bed (LiCor). The consensus binding sequence for AP-1 was 5′-CGCTTGATGACTCAGCCGGAA-3′.

#### Laser scanning confocal microscopy

Primary monocytes were isolated according to the protocol described above. Fresh cells were plated on coverslips (Nest) and fixed in 4% paraformaldehyde (Beyotime)/PBS at RT for 30 min, permeabilized with 0.5% Triton X-100 (Beyotime) /PBS at RT for 10 min, followed by blocking with PBS containing 1% bovine serum albumin (BSA) and 1% rabbit serum for 30 min. Fixed cells were incubated with primary antibody against CYP1A1 (1:250, CST), phosphorylated JNK (p-JNK, 1:100, CST), phosphorylated c-jun (1:100, CST) and phosphorylated c-fos (1:100, CST) at 4 °C overnight, followed by Alexa Fluor 594-conjugated donkey secondary antibody (1:200, Invitrogen) in the dark for 2 h in PBS containing 1% BSA and 0.5% Triton X-100. Cells were then counterstained with DAPI (Beyotime) for 10 min. After incubation, all coverslips were washed three times in PBS and imaged using a LSM 780 laser-scanning confocal microscope (Carl Zeiss).

### Inhibitor assays

#### In vitro

RAW264.7 cells were seeded at 2 × 10^5^ cells/well in six-well plates and cultured for 24 h in RPMI 1640. PMs were seeded at 1 × 10^6^ cells/well. Cells were then pre-treated with vehicle, 12(S)-HETE antibody (3 *μ*g/ml, 12 h before challenge), ML355 (10 *μ*M, 2 h before challenge) or SR-A monoclonal antibody (3 *μ*g/ml, 12 h before challenge) as indicated then treated with vehicle, LPS (10 *μ*g/ml) or *E.coli* (MOIs=30) for the indicated times.

#### In vivo

C57BL/6 mice (male, 10−12 weeks, 20−25 g) were fasted for 12 h and then pre-treated with vehicle, JNK inhibitor (30 mg/kg) or AP-1 inhibitor (20 mg/kg) for 2 h. Mice were then treated with vehicle or *E. coli* (1.2 × 10^11^ CFUs/kg) for the indicated times. In survival experiments, JNK and AP-1 inhibitors were re-injected 6, 12 and 24 h after *E. coli* or CLP challenge. Plasma was isolated from mouse venous blood for various markers assays as described.

### CYP1A1 hydroxylase activity assay

Benzo[*α*]pyrene is frequently used as a substrate for assessing CYP1A1 hydroxylase activity. The measurement was consulted with previous studies [26, 27].

### Macrophages infection with adenovirus

PMs were seeded at 2 × 10^6^ cells per well on six-well plates and infected with 1000 MOIs negative control adenovirus, adenovirus encoding CYP1A1−GFP or adenovirus encoding a hydroxylase-deficient CYP1A1−GFP for 8 h, then cultured for 40 h in fresh RPMI 1640. After 48 h infection, the macrophages were harvested for Western blot analysis.

### Sepsis models

CLP was applied to induce the polymicrobial sepsis model as previous study described [28]. Mice were injected intraperitoneally with 1.2 × 10^11^ CFU/kg *E. coli* to induce the monomicrobial sepsis [29]. Vector-infected PMs (5 × 10^6^ cells per mouse) were injected intraperitoneally into WT mice 2 days and Rhapontigenin were injected intraperitoneally into WT mice 1 h before *E. coli*- or CLP-induced sepsis and survival monitored for the indicated periods. Some treated mice were sacrificed at 12 h after *E.coli* or CLP challenge for sample collection. PLF was prepared by a single washing of the peritoneal cavity with 3 ml cold PBS. PLF, Spleen and liver were harvested for bacterial burden detection. The 12(S)-HETE, TNF-α and IL-6 concentrations were determined by ELISA and bacterial burden was measured by bacterial colony count in PLF. Serum from indicated septic mice was also extracted for ALT and AST assays. In phagocytosis experiments, vector-infected PMs were injected intraperitoneally into WT mice 30 minutes before *E. coli* onset or 0 minute before CLP onset. Some treated mice were sacrificed at 40 minutes after *E.coli* or CLP challenge for macrophages SR-A mRNA and intracellular bacteria count measurements.

### Detection of bacterial burden

*In vivo* experiments, isopyknic PLFs (3 ml for each), spleen and liver were collected from indicated septic mice after 12 h *E.coli* or CLP challenge. PLFs were diluted 1000-fold with 1 ml sterile LB. Equal amounts of tissues were homogenized in 3 ml sterile LB in ice and lapping tissues fluids were diluted 500-fold with 1.5 ml sterile LB. CYP1A1-modified, Rhapontigenin-treated PMs, extracted PMs from Ad-CYP1A1 macrophages transferred or Rhapontigenin treated septic mice were harvested after 40 minutes impact. The harvested cells were centrifuged for 5 min at 300 g and suspended with 1 ml sterile LB. The suspensions were diluted 100-fold with 1 ml sterile LB. Each dilution was plated on LB agar plate at 200 *μ*l volume respectively and incubated at 37 °C under sterile air atmosphere for 18 h. The numbers of bacterial colonies were calculated as CFUs.

### Study subjects

From November 2017 to December 2017 and November 2018 to December 2018, thirty eligible patients were screened within 48 h after admission to the ICU of the Third Affiliated Hospital of Army Medical University. Patients were enrolled if they had known or suspected infection plus a SOFA score of 2 or more for organ dysfunction and the ability to provide informed consent. Exclusion criteria were age younger than 17 or older than 76 years, mental disorders, or sepsis combined with other severe somatic diseases (malignant tumour or cancer). The clinical data are summarized in supplemental table 2. Thirty healthy individuals in the medical examination centre of the Third Affiliated Hospital of Army Medical University were also enrolled as controls. The study protocol was approved by the local institutional review board, and informed consent was obtained from all subjects or their surrogates. Fresh plasma was isolated from peripheral venous blood (patients were fasted) on day 2 of ICU admission. Monocytes were prepared as previously described. Expression levels of CYP1A1 mRNA were assessed using qRT-PCR. The plasma (diluted 1:100 in assay buffer) concentration of 12(S)-HETE was determined by ELISA. Expression levels of CYP1A1, phosphorylation levels of JNK and AP-1 in monocytes were observed using laser scanning confocal analysis.

### Statistical analysis

Most data are presented as the mean ± standard error (SEM) of three independent experiments. Multiple group means were compared by one-way ANOVA followed by LSD multiple-comparison tests. Survival rates were analysed using the Mantel-Cox test. Categorical data were compared between two groups by chi-squared test. Continuous variables were compared between two groups by two-tailed Student′s *t*-test. Correlations with SOFA scores were analysed by linear regression. All statistical analyses were performed using SPSS 16.0 (SPSS Inc.) or Prism 6.0 (GraphPad software Inc.) software, and a P-value < 0.05 was considered statistically significant.

## Results

### CYP1A1 is upregulated in PMs of septic mice

**Fig. 1:**
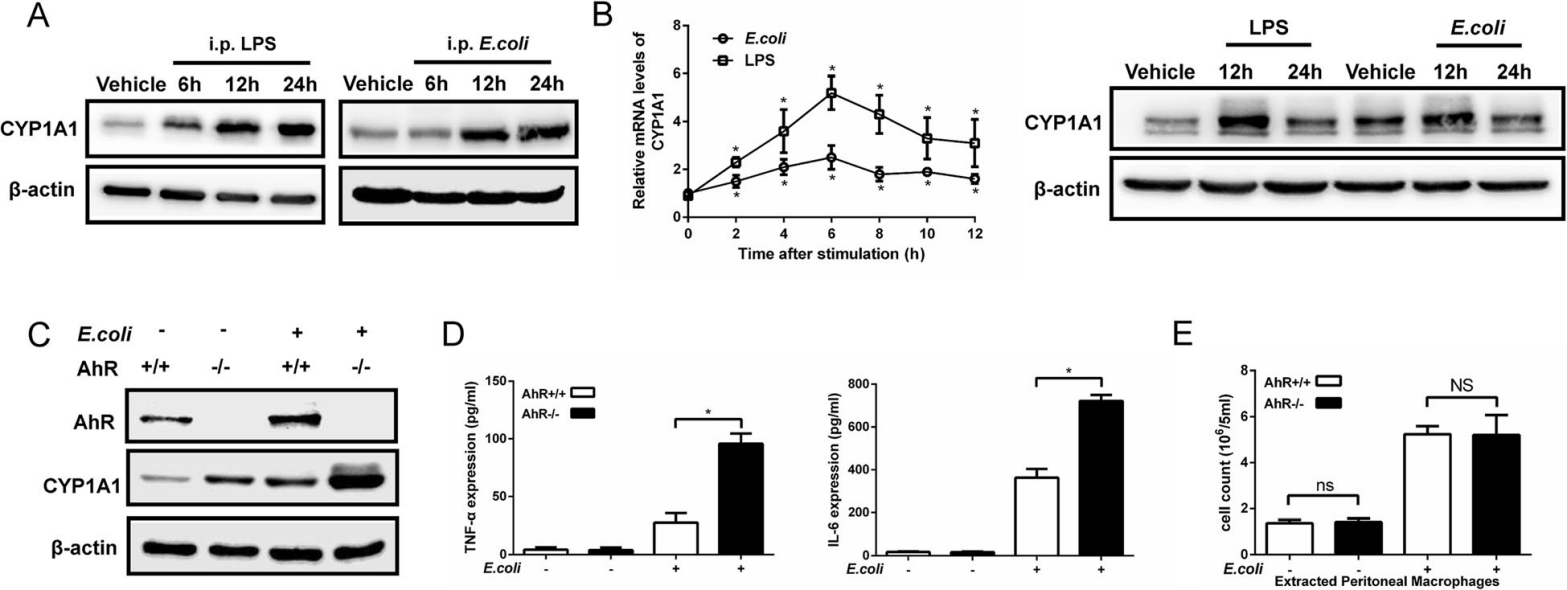
CYP1A1 is upregulated in PMs of septic mice. **a** Mice were intraperitoneally injected with vehicle (isopyknic PBS), LPS (20 mg/kg) or *E. coli* (1.2 × 10^11^ CFUs/kg, CFUs, colony forming units). PMs were extracted at the indicated times and subjected to western blotting analysis of CYP1A1 protein levels. **b** PMs isolated from WT mice were treated with vehicle, LPS (10 μg/ml) or heat-killed *E. coli* (MOIs = 10, MOIs, multiplicity of infections) for the indicated times. CYP1A1 mRNA expression was quantified by qRT-PCR. Expression levels of CYP1A1 protein were detected by western blotting. **c**-**e** AhR^−/−^ and WT mice were intraperitoneally injected with vehicle or *E.coli*. After 12 h treatment, PMs and PLFs were extracted and subjected to analysis of AhR and CYP1A1 protein expression levels (**a**), pro-inflammatory cytokines expression levels (**b**) and PMs count (**c**). Data are mean ± SEM of three independent experiments. Results were compared by one-way ANOVA. *p < 0.05. NS, no statistical difference

In current study, we found that CYP1A1 was significantly elevated in PMs isolated from LPS- or *E. coli*-induced mice (Fig. 1a). The levels of CYP1A1 mRNA in PMs were elevated at 2 h and reached a peak at 6 h after heat-killed *E. coli* or LPS treatment, while CYP1A1 protein expression was significantly upregulated at 12 h after challenge (Fig. 1b). AhR is the main regulator of CYP1A1 expression [30]. We found that CYP1A1 protein levels were still highly overexpressed in PMs from *E.coli*-treated AhR knockout (AhR^−/−^) mice (Fig. 1c), accompanied by TNF-α and IL-6 in peritoneal lavage fluids (PLFs) increase (Fig. 1d). However, there were no differences in PMs counts between *E.coli*-treated AhR^−/−^ mice and *E.coli*-treated wild-type (WT) mice (Fig. 1e). These results suggest a possible AhR-independent regulation of CYP1A1 in the progression of inflammation and sepsis.

### CYP1A1 is involved in LPS-induced macrophages activation

**Fig. 2:**
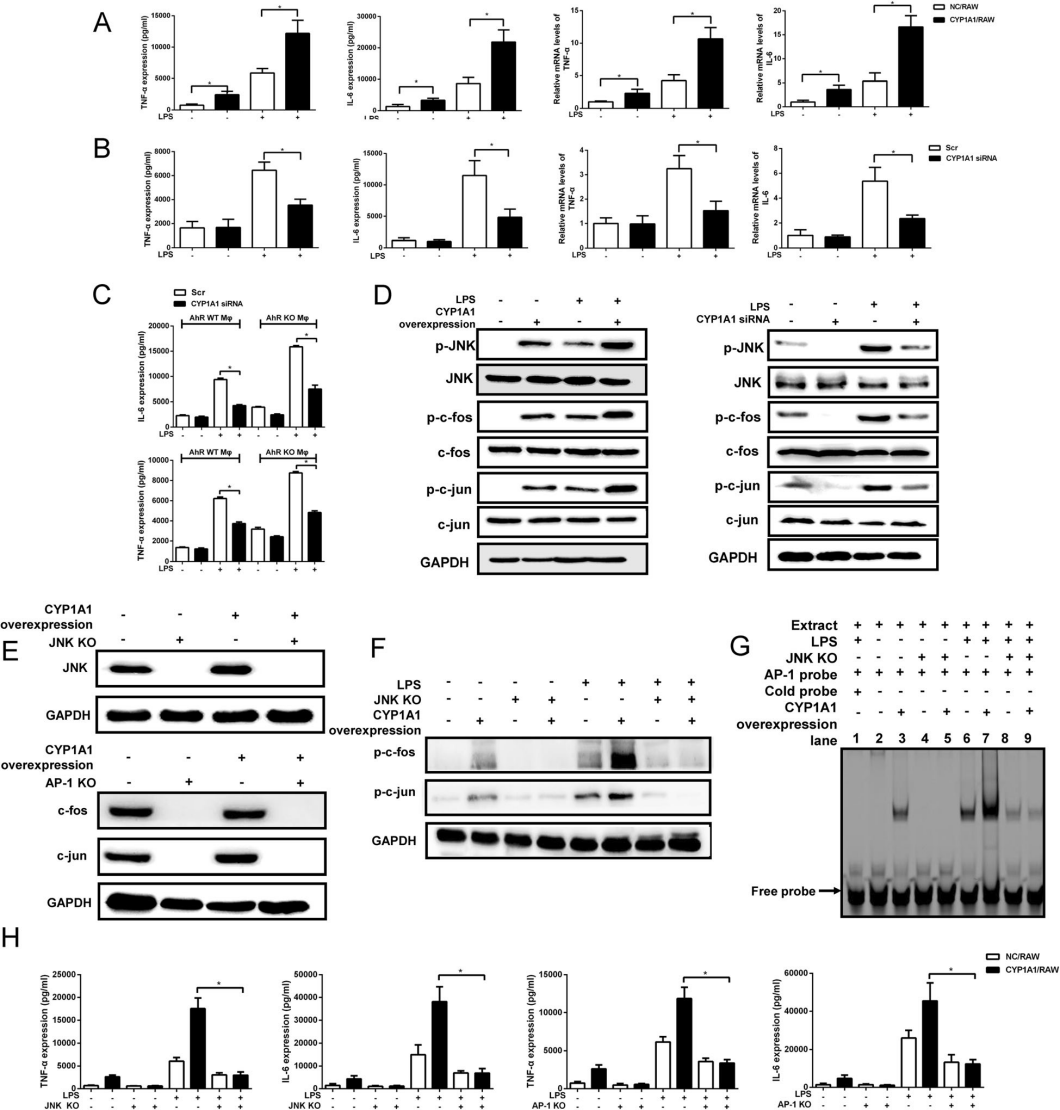
CYP1A1 is involved in LPS-induced macrophages activation. **a**, **d** CYP1A1/RAW and NC/RAW were treated with LPS (10 *μ*g/ml). TNF-α and IL-6 mRNA levels were detected by qRT-PCR after 4 h LPS stimulation. TNF-α and IL-6 protein levels were measured by ELISA after 12 h LPS stimulation. JNK/AP-1 (c-fos and c-jun) activities were assessed by western blotting after 2 h LPS stimulation. **b**, **d** PMs transfected with CYP1A1 siRNA or scramble siRNA were stimulated with LPS. TNF-α and IL-6 mRNA (LPS 4 h) and protein (LPS 12 h) levels and JNK/AP-1 activities (LPS 2 h) were determined respectively. **c** CYP1A1 siRNA or scramble siRNA were transfected into WT and AhR^−/−^ PMs respectively. Transfected cells were stimulated with LPS for 12 h. Supernatants were collected for analysis of TNF-α and IL-6 protein release using ELISA. **e** JNK and AP-1 protein levels were detected in CYP1A1/RAW and NC/RAW in which JNK and AP-1 were knocked out. **f**, **g** JNK-knockout CYP1A1/RAW and NC/RAW were treated with vehicle or LPS for 2 h. **f** The cells were lysed for western blotting analysis of phosphorylated c-fos and c-jun. **g** The nuclear extract proteins of treated cells were incubated with AP-1-binding site probe and binding activity measured by EMSA. **h** JNK-knockout CYP1A1/RAW and NC/RAW, as well as AP-1 knockout-CYP1A1/RAW and NC/RAW were stimulated with LPS for 12 h and supernatants were collected for analysis of TNF-α and IL-6 protein release using ELISA. Data are mean ± SEM of three independent experiments. Results were compared by one-way ANOVA. *p < 0.05

To directly assess the function of CYP1A1 in regulating inflammatory factor expression, we established a cell line derived from RAW constitutively expressing CYP1A1 (CYP1A1/RAW) and a negative control line (NC/RAW) (Supplemental Figure 1 A). These CYP1A1/RAW exhibited significantly greater expression of TNF-α and IL-6 at both mRNA and protein levels following LPS challenge than LPS-treated NC/RAW (Fig. 2a), while expression levels of IL-1β and NOS2 mRNA were not altered. We found that mRNA levels of Arg-1 and PPARγ, two markers of M2 macrophage, were reduced in LPS-stimulated CYP1A1/RAW compared with LPS-stimulated NC/RAW (Supplemental Figure 2), suggesting that CYP1A1 may inhibit M2 macrophage polarization during LPS-induced inflammation. To confirm these regulatory effects of CYP1A1 on TNF-α and IL-6 expression, we also examined LPS responses in macrophage transfected with a CYP1A1-targeting short interfering RNA (CYP1A1 siRNA) or scramble siRNA (Scr control). As CYP1A1 expression levels are too low to implement gene silencing in RAW, we instead conducted these experiments in mice PMs (Supplemental Figure 1 B). Consistent with overexpression experiments, expression levels of IL-6 and TNF-α were reduced in CYP1A1 knockdown PMs following LPS treatment (Fig. 2b). Moreover, pre-treatment of PMs with a selective CYP1A1 enzymatic inhibitor Rhapontigenin [31, 32] following by LPS stimulation reduced IL-6 and TNF-α levels compared to vehicle pre-treatment (Supplemental Figure 3). Interestingly, we found that LPS-stimulated TNF-α and IL-6 expression levels were still lower in CYP1A1 silent AhR^−/−^ PMs compared to Scr control transfected AhR^−/−^ PMs, suggesting that CYP1A1-induced pro-inflammatory responses are also AhR-independent (Fig. 2c).

MAPK/AP-1 and NF-κB are two main pathways of TNF-α and IL-6. Overexpression of CYP1A1 significantly enhanced phosphorylation of AP-1 and its upstream regulator JNK following LPS, while CYP1A1 knockdown reduced these phosphorylation responses (Fig. 2d). Alternatively, there were no differences in phosphorylation levels of p-38 between LPS-stimulated CYP1A1/RAW and NC/RAW, while the phosphorylation levels of NF-κB and ERK_1/2_ exhibited a moderate decrease in LPS-induced CYP1A1/RAW compared to LPS-induced NC/RAW (Supplemental Figure 4). To directly demonstrate the role of JNK/AP-1 in CYP1A1-associated TNF-α and IL-6 expression, we knocked out JNK and AP-1 in NC/RAW and CYP1A1/RAW, respectively (Fig. 2e). LPS-induced AP-1 phosphorylation was decreased in JNK knockout CYP1A1/RAW compared whit control CYP1A1/RAW (Fig. 2f). The DNA-binding activity of AP-1 was also greatly reduced by JNK knockout (Fig. 2g). Likewise, LPS-stimulated TNF-α and IL-6 expression levels were lower in both JNK and AP-1 knockout CYP1A1/RAW compared with control CYP1A1/RAW (Fig. 2h). Collectively, these data strongly suggest that CYP1A1 upregulates LPS-induced TNF-α and IL-6 expression by strengthening JNK-mediated AP-1 activation.

### CYP1A1-derived 12(S)-HETE production intensifies JNK/AP-1 activation

**Fig. 3:**
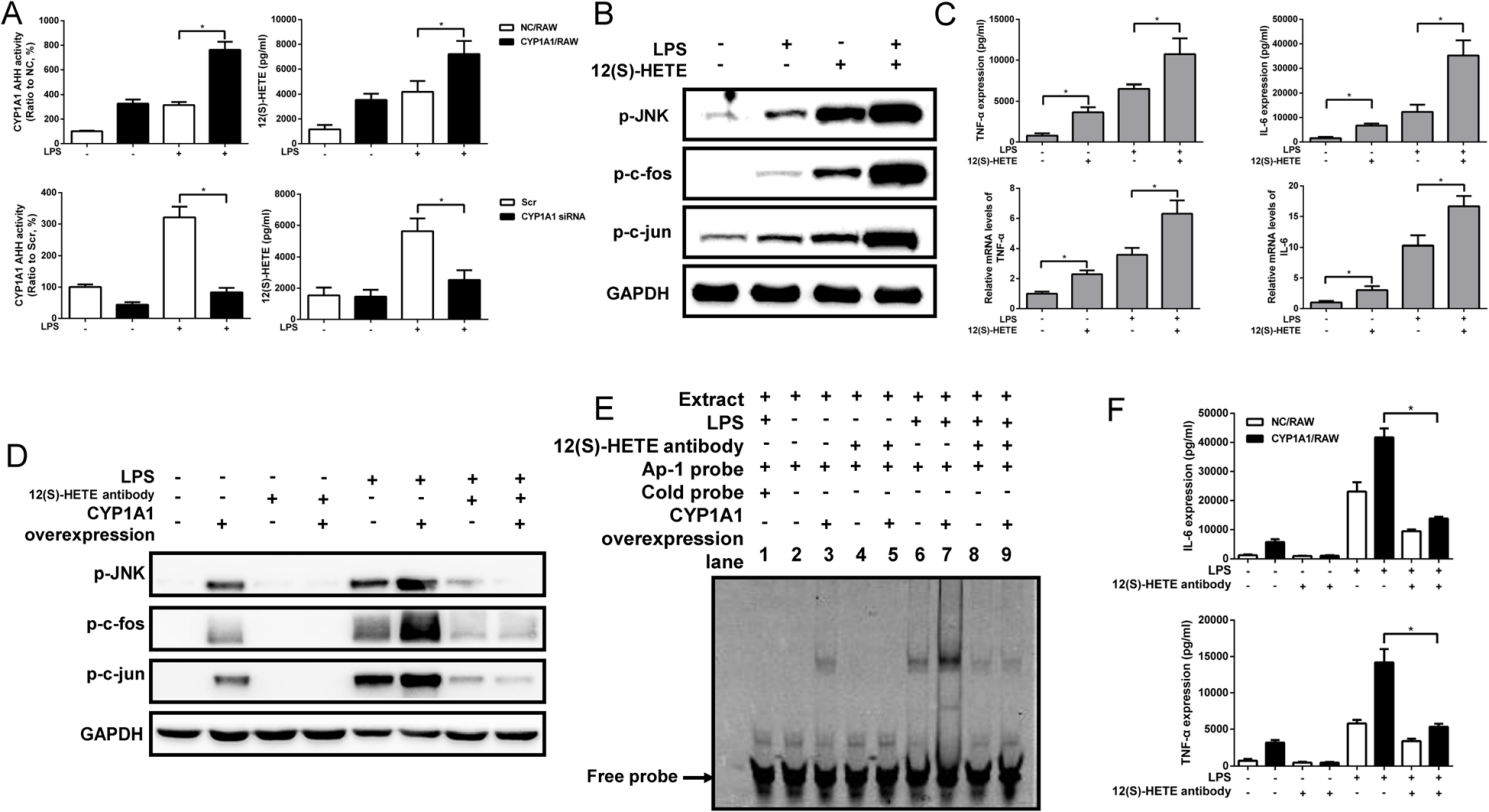
CYP1A1-derived 12(S)-HETE production intensifies JNK/AP-1 activation. **a** CYP1A1/RAW and NC/RAW, as well as PMs transfected with CYP1A1 siRNA or scramble siRNA were treated with vehicle or LPS (10 *μ*g/ml) for 12 h, and supernatants were then assayed for 12(S)-HETE concentration and CYP1A1 hydroxylase activity (AHH; presented as %) by ELISA and a standard AHH activity protocol, respectively. **b**, **c** RAW cells were treated with vehicle or LPS and 12(S)-HETE (500 nM) for 2 h (**b**) and 12 h (**c**). **b** The cell lysates were used for western blotting analysis of JNK/AP-1 phosphorylation levels. **c** TNF-α and IL-6 protein levels were assessed by ELISA. **d**-**f** CYP1A1/RAW and NC/RAW were administrated 12(S)-HETE antibody (3 *μ*g/ml) for 12 h and then LPS for 2 h (western blotting and EMSA) and 12 h (ELISA). **d** Treated cells were lysed for measuring JNK/AP-1 phosphorylation level by western blotting. **e** Nuclear-extract proteins of treated cells were incubated with AP-1-binding site probe and binding activity measured using EMSA. **f** TNF-α and IL-6 protein levels were assessed by ELISA. Data are shown as mean ± SEM of three independent experiments. Results were compared by one-way ANOVA. *p < 0.05

12(S)-HETE can activate AP-1 [22–24], so we further examined if CYP1A1 promotes LPS-induced inflammatory responses through 12(S)-HETE. Our data showed there is a positive correlation between CYP1A1 and 12(S)-HETE production, which is associated with CYP1A1 hydroxylase activity (Fig. 3a). Moreover, direct treatment of RAW with 12(S)-HETE and LPS resulted in greater JNK/AP-1 phosphorylation and TNF-α and IL-6 expression compared to vehicle- and LPS-treated control RAW (Fig. 3b and c). To further confirm that the inflammatory response caused by CYP1A1 overexpression is mediated by 12(S)-HETE, we used a 12(S)-HETE-blocking antibody to neutralize 12(S)-HETE in LPS-stimulated CYP1A1/RAW and NC/RAW. Indeed, this treatment abolished JNK/AP-1 activation and TNF-α and IL-6 elevation associated with CYP1A1 overexpression (Fig. 3d-f). These results suggest that CYP1A1 controls the activation of macrophages in response to LPS via 12(S)-HETE generation.

### Elevation of 12(S)-HETE in LPS-stimulated CYP1A1/RAW cells is CYP1A1 hydroxylase-dependent rather than 12 lipoxygenase-dependent

**Fig. 4:**
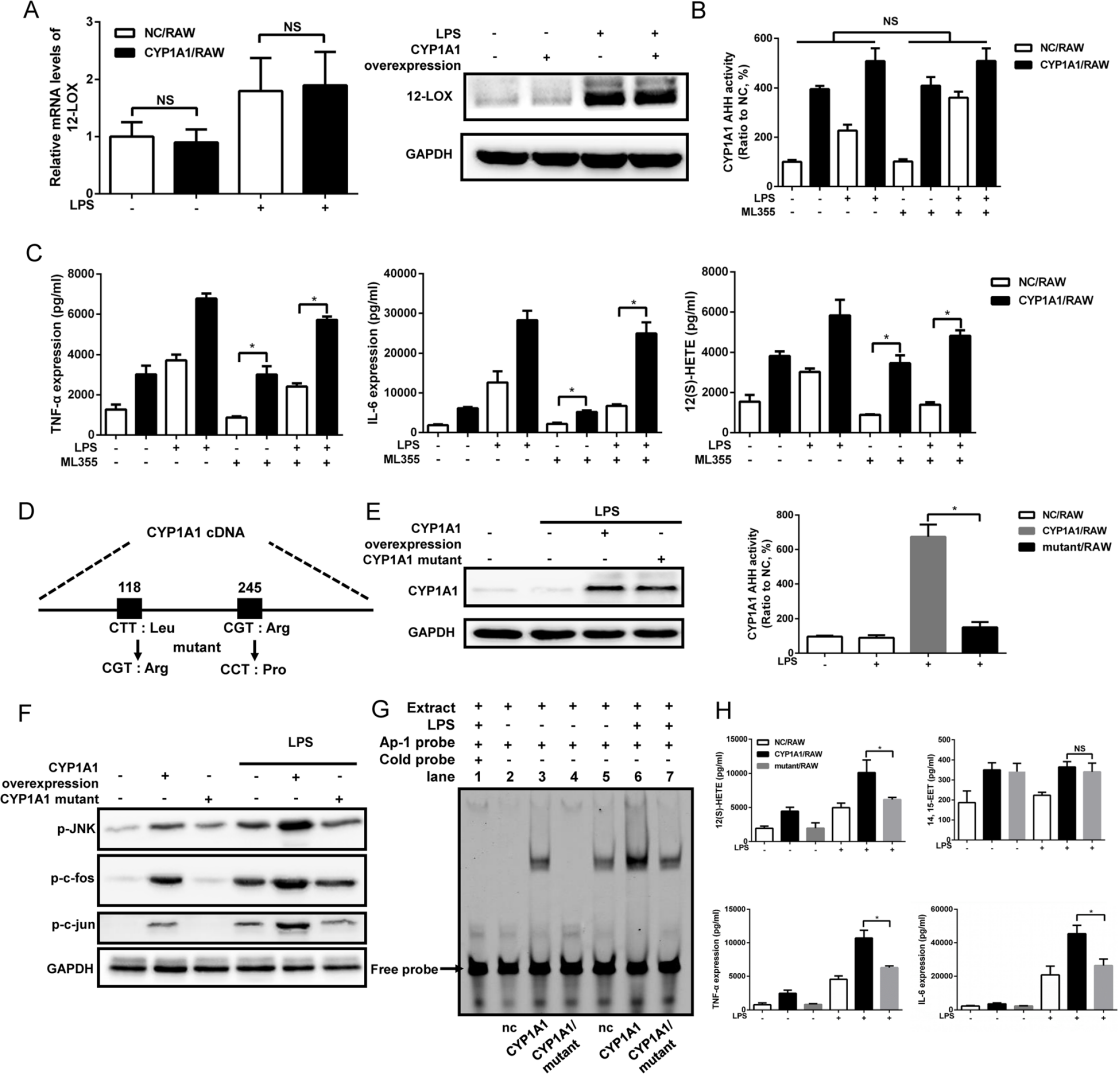
Elevation of 12(S)-HETE in LPS-stimulated CYP1A1/RAW cells is CYP1A1 hydroxylase-dependent rather than 12 lipoxygenase-dependent. **a**, **b** CYP1A1/RAW and NC/RAW were stimulated with vehicle or LPS (10 μg/ml) for 2 h for qRT-PCR or 12 h for western blotting. **a** 12-LOX mRNA levels were detected using qRT-PCR. 12-LOX protein levels were assessed by western blotting. **b**, **c** CYP1A1/RAW and NC/RAW were pre-treated with the selective 12-LOX inhibitor ML355 (10 μM) for 2 h and then stimulated with LPS for 12 h. **b** Supernatants were collected for CYP1A1 AHH activity measurement using a standard AHH activity assay protocol. **c** Alternatively, TNF-α, IL-6 and 12(S)-HETE levels were detected in supernatants using ELISA. **d** Schematic of CYP1A1 cDNA nucleotide sequence containing two mutant positions that impair CYP1A1 AHH activity. **e**, **h** CY1A1/RAW, NC/RAW and CYP1A1 mutant/RAW were treated with LPS for 12 h. **e** CYP1A1 protein levels were measured from cell lysate by western blotting. CYP1A1 AHH activity was measured in supernatants using a standard AHH activity assay. **f**, **g** CY1A1/RAW, NC/RAW and CYP1A1 mutant/RAW were treated with LPS for 2 h. **f** Treated cells were lysed and subjected to western blotting analysis. **g** The nuclear-extract proteins of treated cells were incubated with AP-1-binding site probe and binding activity measured by EMSA. **h** Supernatants were collected for analysis of TNF-α, IL-6, 12(S)-HETE and 14, 15-EET levels using ELISA. Data shown as mean ± SEM of three independent experiments. Results were compared by one-way ANOVA. *p < 0.05. NS, no statistical difference

It was demonstrated that 12(S)-HETE also benefits from 12-lipoxygenase (12-LOX) [33, 34]. However, overexpressed-CYP1A1 failed to affect 12-LOX expression at either the mRNA or protein level (Fig. 4a). Furthermore, treatment of CYP1A1/RAW and NC/RAW with the specific 12-LOX inhibitor ML355 [35] had no effect on CYP1A1 hydroxylase activity (Fig. 4b) and failed to abolish the production of 12(S)-HETE, TNF-α, and IL-6 by CYP1A1/RAW and NC/RAW following LPS stimulation (Fig. 4c). It has been reported that CYP1A1 can metabolise AA to epoxyeicosatrienoic acids (EETs) by epoxidase activity as well as to HETEs by hydroxylase activity [19]. To confirm the pro-inflammatory effect caused by CYP1A1 was mainly due to its hydroxylase activity, we established a RAW cell line constitutively expressing a hydroxylase-deficient CYP1A1 (CYP1A1 mutant/RAW) according to previous research [32] (Fig. 4d). Disruption of CYP1A1 hydroxylase activity had no effect on CYP1A1 protein expression (Fig. 4e). 14, 15-EET, a representative EET produced by CYP1A1 [19], was not affected by CYP1A1 hydroxylase deficiency (Fig. 4h), indicating the mutant form we used is hydroxylase-specific. However, JNK/AP-1 phosphorylation and AP-1 − DNA binding activity were reduced in CYP1A1 mutant/RAW compared with normal CYP1A1/RAW after LPS stimulation (Fig. 4f and g). Furthermore, secretion of 12(S)-HETE, TNF-α and IL-6 were reduced (Fig. 4h). Collectively, these findings indicate that CYP1A1 upregulates the LPS-induced inflammatory response of macrophages through concomitant overproduction of 12(S)-HETE via CYP1A1 hydroxylase activity rather than 12 lipoxygenase activity.

### Regulation of the CYP1A1–12(S)HETE−JNK − AP-1 signalling axis in septic mice

**Fig. 5:**
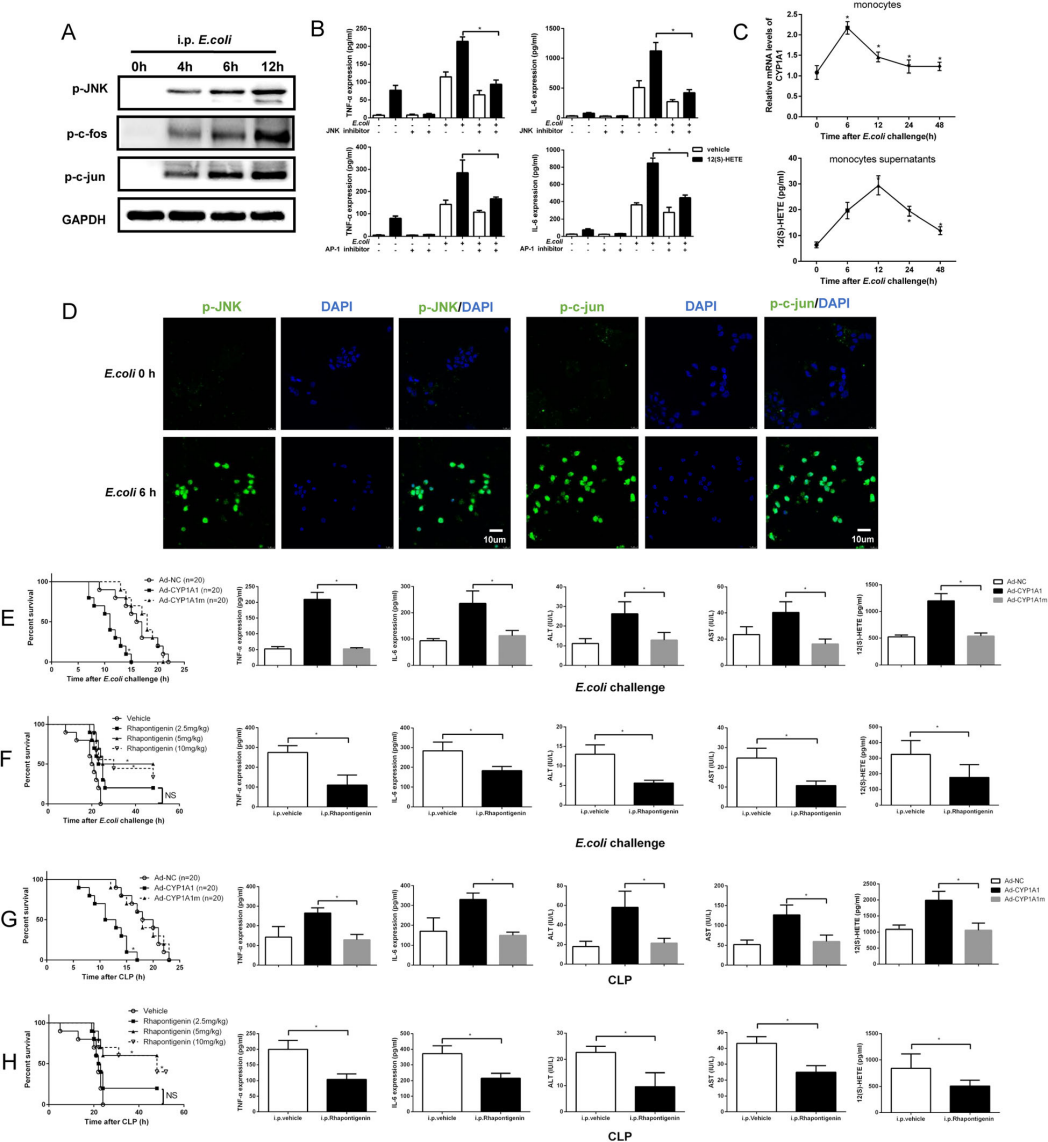
Regulation of the CYP1A1–12(S)HETE−JNK − AP-1 signalling axis in septic mice. **a** Mice were intraperitoneally injected with *E. coli* (1.2 × 10^11^ CFUs/kg) at the indicated times, respectively. PMs were isolated from *E. coli-*treated mice and lysed for analysis of JNK/AP-1 phosphorylation. **b** Mice were pre-treated with JNK inhibitor (30 mg/kg), AP-1 inhibitor (20 mg/kg), or vehicle for 2 h and then stimulated with *E. coli* and 12(S)-HETE (10 mg/kg) for 12 h. PLFs were extracted for analysis of TNF-α and IL-6 protein levels (*n* = 6). Results were compared by one-way ANOVA. **c**, **d** Mice were intraperitoneally injected with *E. coli*. **c** Peripheral monocytes were isolated at the indicated times and cultured for 2 h. Supernatants were collected for analysis of 12(S)-HETE levels using ELISA. Results were compared by one-way ANOVA. CYP1A1 mRNA levels in peripheral monocytes from *E. coli-*treated mice were measured by qRT-PCR. Results were compared by one-way ANOVA. **d** JNK/c-jun phosphorylation in peripheral monocytes from *E. coli-*treated mice was assessed using LSCM. Bar: 10 μm. **e**, **g** PMs transfected with Ad-NC, Ad-CYP1A1 or Ad-CYP1A1m were injected intraperitoneally into WT mice 2 days before *E. coli* or CLP impact. **f**, **h** Rhapontigenin (2.5 mg/kg, 5 mg/kg and 10 mg/kg dosages were used in this experiment) were intraperitoneally injected into WT mice 1 h before *E. coli* or CLP impact. **e**-**h** Survival rates were monitored for 2 days after *E. coli* or CLP impact and presented as Kaplan-Meier survival curves. Results were compared by log-rank test (*n* = 20). PLFs and peripheral serum were collected from each group 12 h after *E. coli* or CLP impact and TNF-α (PLFs), IL-6 (PLFs), 12(S)-HETE (PLFs), ALT (serum) and AST (serum) levels were detected by ELISA (*n* = 6). Results were compared by one-way ANOVA. *p < 0.05. NS, no statistical difference

After CLP or *E. coli* impact, we found that the levels of 12(S)-HETE were elevated in mice PLFs (Supplemental Figure 5). JNK/AP-1 phosphorylation was also increased in PMs extracted from *E. coli*-challenged WT mice (Fig. 5a). Consistent with in vitro results, intraperitoneal injection of 12(S)-HETE elevated the concentrations of TNF-α and IL-6 in mice PLFs, while JNK and AP-1 inhibitors abolished these effects (Fig. 5b). Furthermore, we isolated and cultured the peripheral monocytes from *E. coli-*treated mice to investigate the direct relationship among 12(S)-HETE, CYP1A1 and monocytes. The levels of 12(S)-HETE in monocytes cultured supernatants and levels of CYP1A1 in monocytes were elevated after *E. coli* treatment, respectively (Fig. 5c). The activation of JNK and c-jun in peripheral monocytes from *E. coli-*treated mice was also confirmed by laser scanning confocal microscopy (LSCM) (Fig. 5d).

To specifically assess the involvement of CYP1A1 in sepsis, recombinant CYP1A1-expression adenovirus (Ad-CYP1A1), hydroxylase-deficient CYP1A1 adenovirus (Ad-CYP1A1m) and negative control adenovirus (Ad-NC) were constructed and transfected into WT PMs (Supplemental Figure 6 A) and then evaluated the effects of these cells on the mortality of *E. coli*- or CLP-induced septic WT mice by adoptive cell transfer (Supplemental Figure 6 B). Compared to Ad-NC PMs injection, Ad-CYP1A1 PMs injection significantly accelerated the mortality and increased the expression levels of pro-inflammatory biomarkers, including ALT, AST, TNF-α, IL-6 and 12(S)-HETE, of mice in the *E. coli*- or CLP-induced septic mice (Fig. 5e and g), while Ad-CYP1A1m PMs injection had no these effects. Furthermore, treatment of mice with JNK and AP-1 inhibitors prior to Ad-NC and Ad-CYP1A1 PMs adoptive transfer decreased mice mortality and pro-inflammatory biomarkers in the *E. coli*- or CLP-induced sepsis model (Supplemental Figure 7). On the other hand, intraperitoneal injection of Rhapontigenin increased the survival and decreased pro-inflammatory biomarkers of mice in *E. coli*- or CLP-induced sepsis model (Fig. 5f and h). As an important source of 12(S)-HETE, our data showed that platelet counts are abundantly increased in the peritoneal cavity following LPS, *E.coli*, or sepsis impact, while CYP1A1 overexpression in PMs was incapable to recruit platelets in PLFs (Supplemental Figure 8). Altogether, these findings confirmed the regulatory effects of CYP1A1/12(S)-HETE/JNK/AP-1 axis in vivo, while Rhapontigenin reduces the mortality and pro-inflammatory biomarkers from sepsis.

### CYP1A1 is involved in phagocytosis of bacteria in macrophages during sepsis

**Fig. 6:**
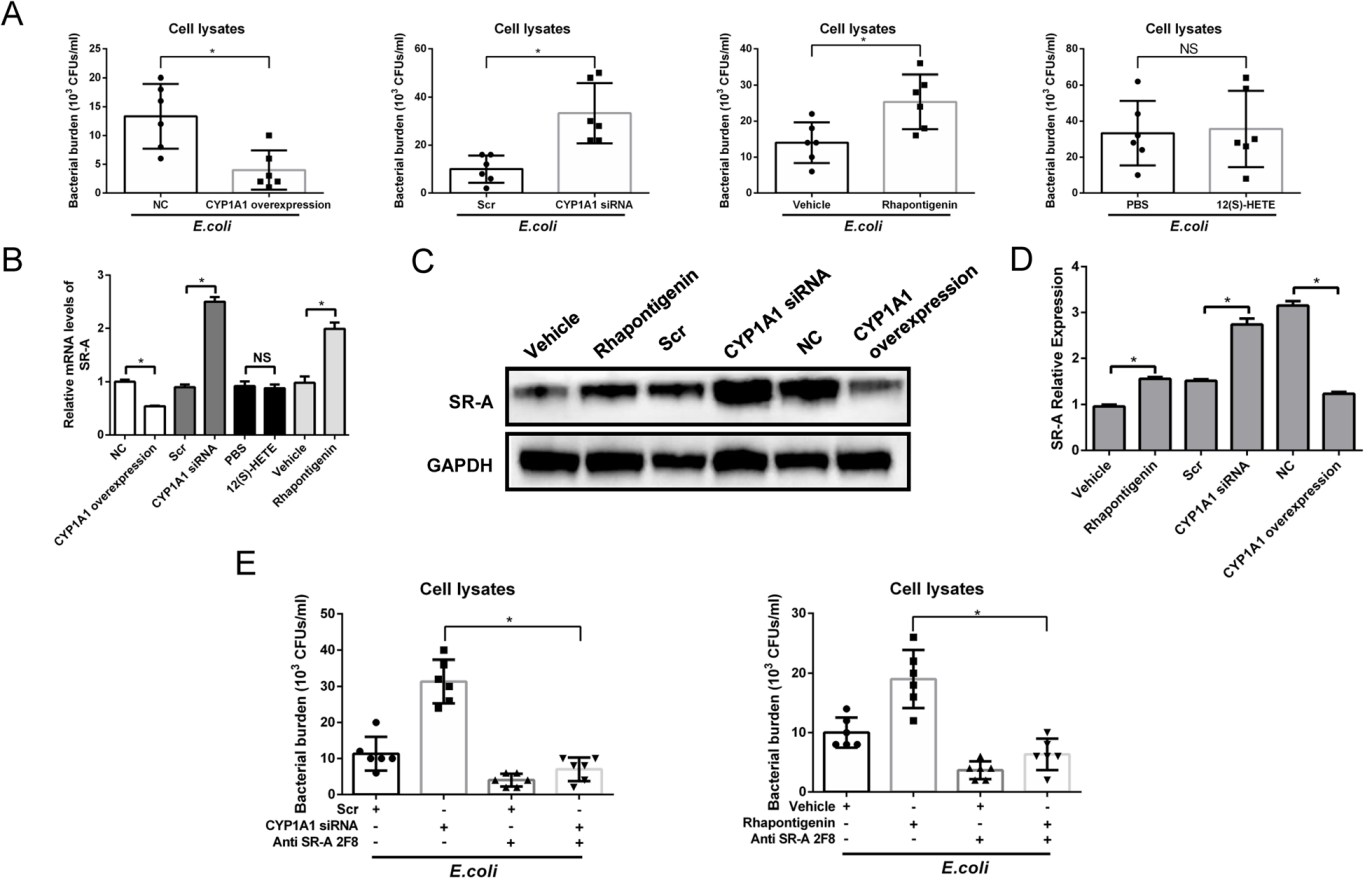
CYP1A1 is involved in phagocytosis of bacteria in macrophages during sepsis. **a**-**d** CYP1A1 overexpression, CYP1A1 knockdown and WT PMs were treated with *E. coli* (MOIs = 30) for 40 min. WT PMs were pre-treated with Rhapontigenin (10 *μ*M) or 12(S)-HETE (500 nM) for 2 h before *E.coli* onset. **a** Cells were washed and subjected to intracellular bacteria count detection. Group means were compared by Student’s *t* test (*n* = 6). **b** Cells were collected for SR-A mRNA measure. Results were compared by one-way ANOVA. Data are shown as mean ± SEM of three independent experiments. **c** Cells were lysed for western blotting analysis of SR-A protein expression. **d** SR-A protein expression was quantified by densitometry using GAPDH as the gel-loading control. Results were compared by one-way ANOVA. **e** CYP1A1 knockdown macrophages and Rhapontigenin-treated WT macrophages were pre-treated with SR-A antibody (3 *μ*g/ml) for 12 h respectively and then cultured with *E.coli* for 40 min. Cells were washed and subjected to intracellular bacteria count detection. Group means were compared by Student’s *t* test (*n* = 6). *p < 0.05. NS, no statistical difference

In current study, we observed a decrease in the count of intracellular bacteria in CYP1A1-overexpressing PMs following 40 min *E.coli* challenge, while the count of intracellular bacteria was increased in CYP1A1 knockdown and Rhapontigenin-treated PMs, respectively. However, direct treatment of PMs with 12(S)-HETE failed to change the count of intracellular bacteria in *E.coli*-treated PMs (Fig. 6a). SR-A is a critical controller of ingesting *E.coli* in macrophages [36]. Overexpression of CYP1A1 decreased the expression level of SR-A following *E.coli* treatment, while CYP1A1 knockdown and Rhapontigenin treatment elevated the expression levels of SR-A. Direct treatment of PMs with 12(S)-HETE had no effect on the expression of SR-A (Fig. 6b-d).

To directly demonstrate the role of SR-A in CYP1A1-invovled bacteria ingestion, we used SR-A antibody in CYP1A1 silent and Rhapontigenin-treated PMs. The count of intracellular bacteria was significantly reduced in SR-A blocked PMs compared with control cells (Fig. 6e). Consistent with in vitro experiments, we observed decreased count of intracellular bacteria and mRNA levels of SR-A in PMs extracted from septic mice transferred with Ad-CYP1A1 PMs (Supplemental Figure 9 A, C), while the count of survival bacteria was elevated in PLFs (Supplemental Figure 9 B, D). In contrast, the count of intracellular bacteria and mRNA levels of SR-A were elevated in PMs extracted from septic mice treated with Rhapontigenin (Supplemental Figure 9 E, G), while the count of survival bacteria was decreased (Supplemental Figure 9 F, H). Taken together, these results indicate that CYP1A1 in macrophages plays a critical role in host defence against invading bacteria during sepsis.

### The CYP1A1–12(S)-HETE-JNK-AP-1 signalling axis is activated in septic patients

**Fig. 7:**
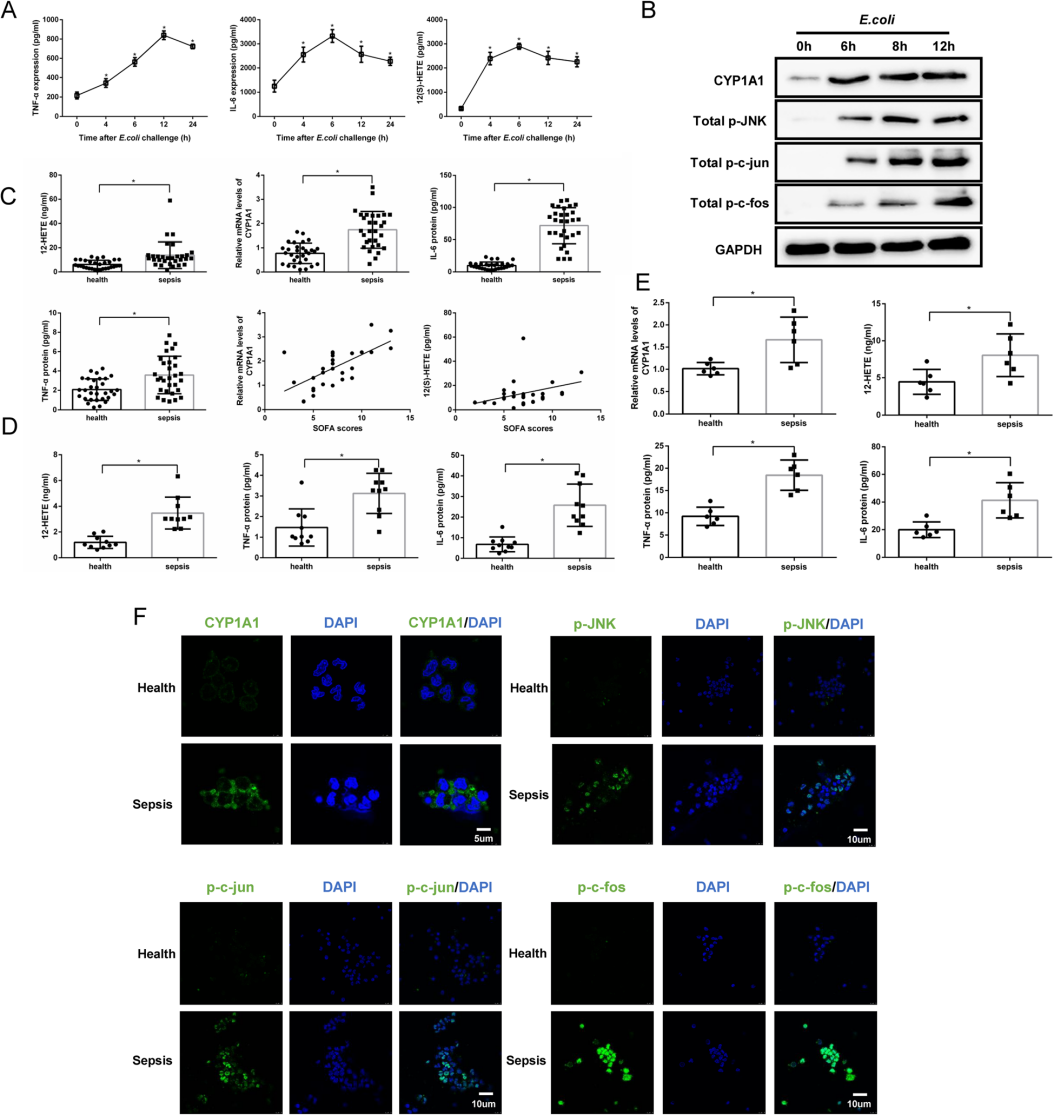
The CYP1A1–12(S)-HETE-JNK-AP-1 signalling axis is activated in septic patients. **a**, **b** Human peripheral monocytes were isolated from healthy donors and treated with heat-killed *E. coli* (MOIs = 10) at the indicated times. Supernatants were collected for analysis of TNF-α, IL-6 and 12(S)-HETE levels using ELISA. Cell lysates were used for JNK/AP-1 phosphorylation assessment. Mean of three independent experiments. Results were compared by one-way ANOVA. **c** CYP1A1 mRNA levels in peripheral monocytes from the studied subjects were measured by qRT-PCR. TNF-α, IL-6 and 12(S)-HETE levels in plasma were analysed by ELISA (*n* = 30 for healthy individuals, *n* = 30 for sepsis patients). Group means were compared by Student’s *t* test. Correlation of SOFA scores with CYP1A1 (r = 0.65, *P* < 0.05) and 12(S)-HETE (r = 0.38, *P* < 0.05) in sepsis patients. **d** Peripheral monocytes were isolated from 10 septic patients and 10 healthy controls and cultured for 2 h, respectively. Supernatants were collected for analysis of TNF-α, IL-6 and 12(S)-HETE levels using ELISA. Group means were compared by Student’s *t* test. **e** Peripheral monocytes were isolated from 6 septic patients and 6 healthy controls and coaxed into macrophages by PMA for 48 h. Supernatants were collected for analysis of TNF-α, IL-6 and 12(S)-HETE levels using ELISA. CYP1A1 mRNA levels in monocytes-derived macrophages were measured by qRT-PCR. Group means were compared by Student’s *t* test. **f** CYP1A1 protein, JNK phosphorylation and AP-1 phosphorylation levels in peripheral monocytes from healthy individuals and septic patients were assessed using LSCM. Bar, CYP1A1: 5 *μ*m, JNK/AP-1: 10 *μ*m. Data shown as mean ± SEM of triplicate experiments. * *p* < 0.05

Peripheral monocytes isolated from healthy individuals treated with heat-killed *E. coli* exhibited elevated levels of TNF-α, IL-6 and 12(S)-HETE (Fig. 7a). CYP1A1 protein expression levels as well as JNK and AP-1 phosphorylation levels were also increased (Fig. 7b). We then compared levels of these signalling factors between septic patients and healthy controls (*n* = 30 in each group). Consistent with preclinical results in mice, CYP1A1 mRNA levels in isolated peripheral monocytes, TNF-α, IL-6 and 12(S)-HETE concentrations in peripheral blood plasma were significantly elevated in septic patients compared with healthy controls. More strikingly, both CYP1A1 mRNA expression levels and 12(S)-HETE concentrations in patients were significantly correlated with SOFA score (Fig. 7c). TNF-α, IL-6 and 12(S)-HETE concentrations were also increased in peripheral monocytes (from 10 healthy and 10 septic individuals) cultured supernatants (Fig. 7d). Additionally, monocytes from 6 septic patients and 6 healthy controls were coaxed into macrophages by PMA. The elevations of CYP1A1 in monocytes-derived macrophages, 12(S)-HETE, TNF-α and IL-6 in supernatants were confirmed by RT-qPCR and ELISA, respectively (Fig. 7d). Furthermore, LSCM confirmed that CYP1A1 protein expression and JNK/AP-1 phosphorylation were enhanced in peripheral monocytes of sepsis patients (Fig. 7f). These results suggest that the CYP1A1–12(S)-HETE−JNK − AP-1 signalling axis is also hyperactivated in sepsis patients and contributes to sequential organ failure.

## Discussion

In this study, we found that CYP1A1 expression was increased in PMs activated by either LPS or heat-killed *E. coli*. Surprisingly, upregulation of CYP1A1 in LPS- or *E. coli*-induced PMs was AhR-independent. The expression levels of CYP1A1 have been measured in multiple organs and cells of different inflammatory models. A previous study found that CYP1A1 mRNA levels were markedly elevated in microglia at 12 h after LPS treatment [37]. CYP1A1 protein levels were also increased in mouse lung and liver following incense smoke challenge [13]. Diesel exhaust particles also increased CYP1A1 mRNA expression in human bronchial epithelial cells, accompanied by pro-inflammatory factor elevation [11]. Conversely, CYP1A1 expression was suppressed in mice inflammatory Hepa cells [38, 39] and LPS-stimulated bovine mammary epithelial cells [40]. These results suggest that the relationship between CYP1A1 expression and inflammation might be species-, tissue- and cell type-specific. Here, we report for the first time that *E.coli*-induced CYP1A1 upregulation is AhR-independent in PMs, which is consistent with several lines of evidence. For instance, glycine methyl transferase upregulated CYP1A1 [41], while Nrf2, Oct-1 and C/EBP downregulated CYP1A1 [42, 43], but these findings had not been confirmed in inflammatory macrophages. Although AhR is considered a primary regulator of CYP1A1, our findings suggest that CYP1A1 expression in macrophages is under the control of various transcription factors that can induces CYP1A1 overexpression in response to inflammatory stimulation.

CYP1A1 suppressed inflammatory responses in parenchymal cells, pulmonary macrophages and epithelial cells [16, 40, 44, 45], but also increased inflammatory responses in some cases. CYP1A1 was upregulated in lung and liver of incense-challenged mice, accompanied by inflammatory factor increase [13]. Further, inflammatory factor LTB_4_ was markedly reduced in neutrophils extracted from zymosan-challenged CYP1A1 knockout mice [20]. Systemic CYP1A1 overexpression also markedly increased mice mortality following *Citrobacter rodentium* infection [17], which is associated with the dysfunction of FICZ-AhR-IL-22 axis in T_H_17 cells, while we found that macrophage CYP1A1 could induce pro-inflammatory responses in an AhR-independent manner. These studies are in line with our findings, although they focused on different cells or stimuli. In the current study, TNF-α and IL-6 expression levels were increased in CYP1A1-overexpressing RAW compared to NC cells following LPS stimulation, while siRNA-mediated CYP1A1 knockdown in PMs impaired TNF-α and IL-6 upregulation induced by LPS.

Despite the known immune functions of AhR, there had been no systematic study investigating the role of CYP1A1 in innate immune responses. We demonstrate that pro-inflammatory stimuli induce CYP1A1 upregulation independent of AhR and that this upregulation is necessary for the enhanced expression/release of pro-inflammatory factors TNF-α and IL-6. The function of CYP1A1 in the regulation of various inflammatory factors in other immune cell types warrants further investigation. A previous study found that JNK could be activated by an AhR-related CYP1A1 inducer PCB118 in rat thyroid FRTL-5 cells and this resulted in increased TNF-α and IL-6 expression [46]. Several studies have also found that AP-1 can be activated by various CYP1A1 inducers including TCDD, benzo [*a*] pyrene [25, 47] beta-naphthoflavone [48]. These results are in support of our findings that CYP1A1-overexpression elevated the phosphorylation levels of JNK/AP-1 and resulted in increased TNF-α and IL-6 expression, while JNK knockout impeded AP-1 phosphorylation and pro-inflammatory factor release in macrophages. AP-1 activation appears to be modulated mainly by MAPK signalling pathways, but p38 phosphorylation level showed no specific association with AP-1 phosphorylation under either normal or high CYP1A1 expression following LPS stimulation. Alternatively, JNK/AP-1 knockout and MAPK expression in CYP1A1/RAW and NC/RAW cells confirmed that CYP1A1 overexpression increases TNF-α and IL-6 by facilitating the phosphorylation of JNK, which then activates AP-1. Furthermore, we found that upregulated CYP1A1 impacted the phagocytosis of *E.coli* in macrophages via decreasing the expression of SR-A for the first time, while CYP1A1 knock down or Rhapontigenin treatment enhanced the phagocytosis and SR-A expression, suggesting that the macrophage CYP1A1 also plays an important role in host defence via regulating macrophages phagocytosis and SR-A expression.

12(S)-HETE is involved in many immune-related diseases [49–54], trauma [55] and JNK/AP-1 activation [22–24]. In this study, we demonstrated for the first time that levels of 12(S)-HETE were elevated in septic mice and patients, while treatment of mice with 12(S)-HETE caused more severe inflammatory responses than vehicle-treated controls. As another metabolite contributed by the epoxidase activity of CYP1A1, EETs (such as 11,12-EET) act as anti-inflammatories by suppressing NF-κB activation but promoting JNK/AP-1 phosphorylation [56, 57], while 12(S)-HETE acts as a pro-inflammatory factor by intensifying both NF-κB and JNK/AP-1 phosphorylation [22, 23, 58]. These findings may explain the reciprocal effects of CYP1A1 overexpression on NF-κB and JNK/AP-1 since EETs and 12(S)-HETE have opposite effects on NF-κB but both promote JNK/AP-1 activities.

Several limitations of the current study should be addressed. Although we observed that LPS- or *E. coli*-induced CYP1A1 overexpression was AhR-independent, it remains unclear which transcriptional factors mediate CYP1A1 expression in AhR^−/−^overactivated macrophages. We observed that mRNA expression levels of Arg-1 and PPARγ, two M2 phenotype markers, were decreased in LPS-stimulated CYP1A1/RAW cells compared with NC/RAW cells, but the details of this effect remain to be fully characterized. We also revealed that CYP1A1-associated JNK/AP-1 activation was directly regulated by 12(S)-HETE, but the mechanism of this effect remains unknown. Although it was verified that GPR31, a 12(S)-HETE-selective receptor, mediated the function of 12(S)-HETE in dermatosis [58], the specific function of GPR31 needs to be further investigated in inflammatory macrophages. In addition, it is notable that LPS acts as an inflammatory agent via binding to TLR4, while CYP1A1-induced 12(S)-HETE may act through agitating GPR31. They are two different pathways indeed but they collectively participate in the inflammatory responses via targeting JNK/AP-1 axis. The potential interaction between LPS-TLR4 axis and CYP1A1/12(S)-HETE-GPR31 axis needs a further assessment. Nevertheless, our study demonstrates the fundamental importance of CYP1A1 and 12(S)-HETE in overactivated macrophages.

## Conclusions

**Fig. 8:**
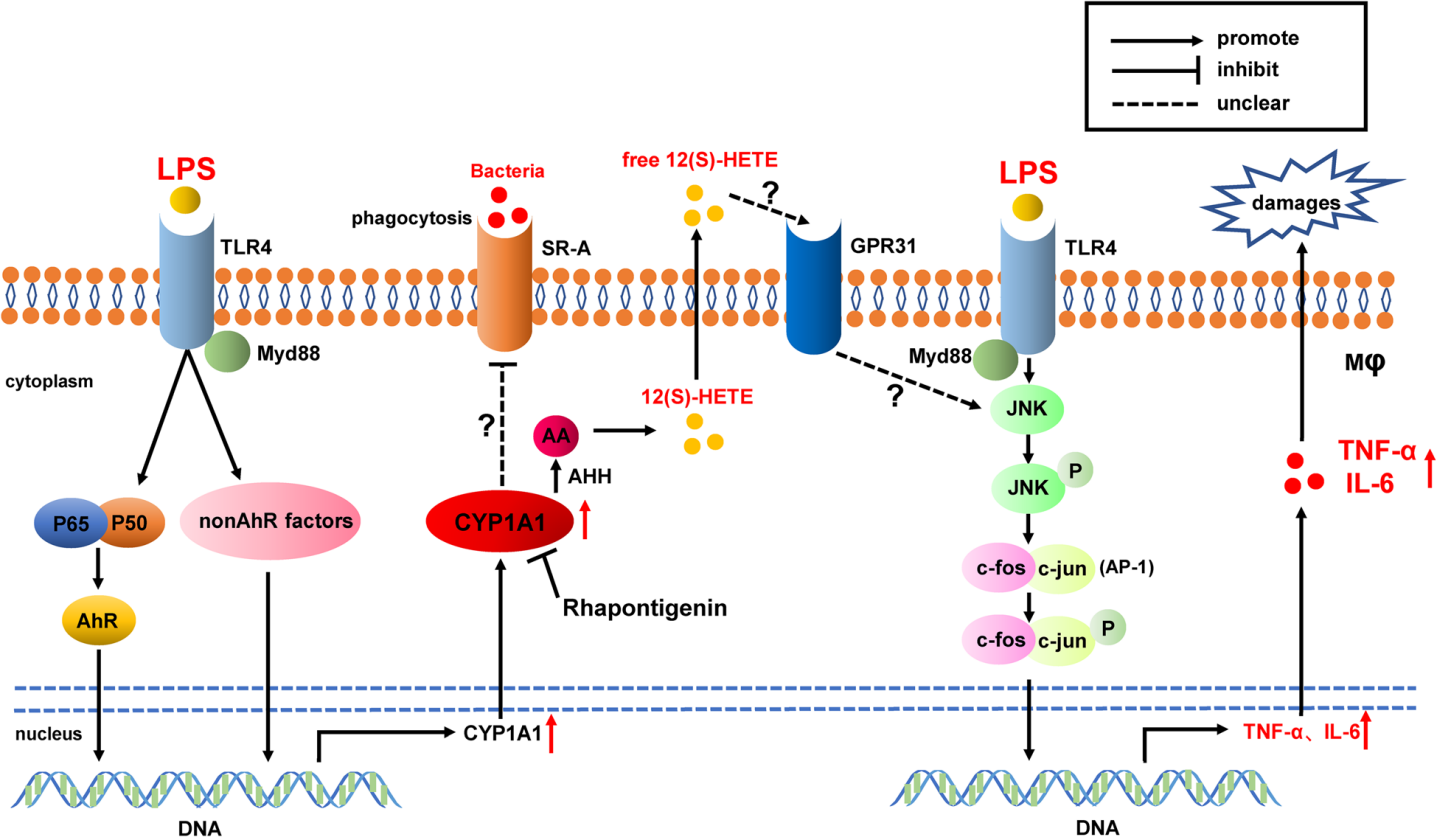
Schematic of the CYP1A1–12(S)-HETE−JNK − AP-1 and CYP1A1-SR-A signalling pathways in macrophage activation. CYP1A1 is highly upregulated in inflammatory macrophages via multiple transcriptional factors. Activated CYP1A1 promotes the production of 12(S)-HETE via its hydroxylase activity and the 12(S)-HETE produced intensifies JNK/AP-1 phosphorylation, which strongly activates genes encoding inflammatory cytokines. Upregulated CYP1A1 also impeded the phagocytosis of *E.coli* by macrophages via depressing SR-A expression. Consequently, these results indicated macrophage CYP1A1 as a negative regulator during septic process and demonstrated that Rhapontigenin could be a novel therapeutic agent against sepsis

In the present study, we showed that CYP1A1 was highly expressed in inflammatory macrophages and promoted TNF-α and IL-6 release. The elevation of TNF-α and IL-6 levels were mediated by JNK/AP-1 activation rather than by the NF-κB pathway. Excessive 12(S)-HETE production mediated by overexpressed CYP1A1 was required for JNK/AP-1 activation in overactivated macrophages. Mice subjected to adoptive transfer of CYP1A1-overexpressing macrophages were more susceptible to fatal *E. coli* challenge, highlighting CYP1A1 as an important driver of inflammatory responses. Moreover, we found positive correlations between SOFA scores and both monocyte CYP1A1 and plasma 12(S)-HETE in septic patients. Collectively, our results identify a novel therapy target, CYP1A1, regulating macrophage inflammatory responses and phagocytosis during sepsis (Fig. 8). Based on these findings, analogues of 12(S)-HETE or selective antagonists of CYP1A1, especially Rhapontigenin, may be the promising treatments against inflammatory diseases and sepsis.

## Supplementary information

**Additional file 1: Figure S1.** Confirmation of transfections. **Figure S2.** Relative expression levels of inflammatory factors in overactivated macrophages. **Figure S3.** The inhibitory effects of Rhapontigenin on LPS-induced TNF-α and IL-6 secretion in PMs. **Figure S4.** Validation of the NF-κB signalling pathway and different MAPK signalling pathways in LPS-stimulated CYP1A1/RAW and NC/RAW. **Figure S5.** The levels of 12(S)-HETE in PLFs from E.coli- and CLP-induced septic mice. **Figure S6.** Detection of lentivirus infection rate in PMs. **Figure S7.** The regulation of CYP1A1-JNK-AP-1 axis in septic mice. **Figure S8.** Platelet count in PLFs from CYP1A1-overexpressed macrophages transferred septic mice. **Figure S9.** CYP1A1 is involved in phagocytosis of bacteria in macrophages during sepsis.

**Additional file 2: Table S1**.

**Additional file 3: Table S2**.

## Data Availability

The datasets used and/or analysed during the current study are available from the corresponding author on reasonable request.

## Abbreviations

12(S)-HETE: 12S-hydroxy-5Z, 8Z, 10E, 14Z-eicosatetraenoic acid
12-LOX: 12-lipoxygenase
AA: Arachidonic acid
AhR: Aryl hydrocarbon receptor
CYP1A1: Cytochrome P450 1A1
EETs: Epoxyeicosatrienoic acids
EMSA: Electrophoretic mobility shift assay
LPS: Lipopolysaccharide
LSCM: Laser scanning confocal microscopy
NC: Negative control
PLFs: Peritoneal lavage fluids
PMs: Peritoneal macrophages
SOFA: Sequential Organ Failure Assessment
SR-A: Scavenger receptor A
