# Correction to: Cytochrome P450 1A1 enhances inflammatory responses and impedes phagocytosis of bacteria in macrophages during sepsis

**DOI:** 10.1186/s12964-020-00597-8

**Published:** 2020-05-18

**Authors:** Li-Xing Tian, Xin Tang, Jun-Yu Zhu, Li Luo, Xiao-Yuan Ma, Shao-Wen Cheng, Wei Zhang, Wan-Qi Tang, Wei Ma, Xue Yang, Chuan-Zhu Lv, Hua-Ping Liang

**Affiliations:** 1 State Key Laboratory of Trauma, Burns and Combined Injury, Department of Wound Infection and Drug, Daping Hospital, Army Medical University, Yuzhong District, Chongqing, China; 2 Trauma Center, The First Affiliated Hospital of Hainan Medical University, Haikou, China; 3 Emergency and Trauma College of Hainan Medical University, Haikou, China

**Correction to: Cell Commun Signal (2020) 18:70**

**https://doi.org/10.1186/s12964-020-0523-3**

Following publication of the original article [1], the authors identified that four western blot bands in Figs. 1, 2 and Additional file 1: Figure S4 were incorrect. The correct images are presented in this correction article and the corrections to these bands do not change the conclusion to the paper. The authors apologize for the error.

**Fig. 1:**
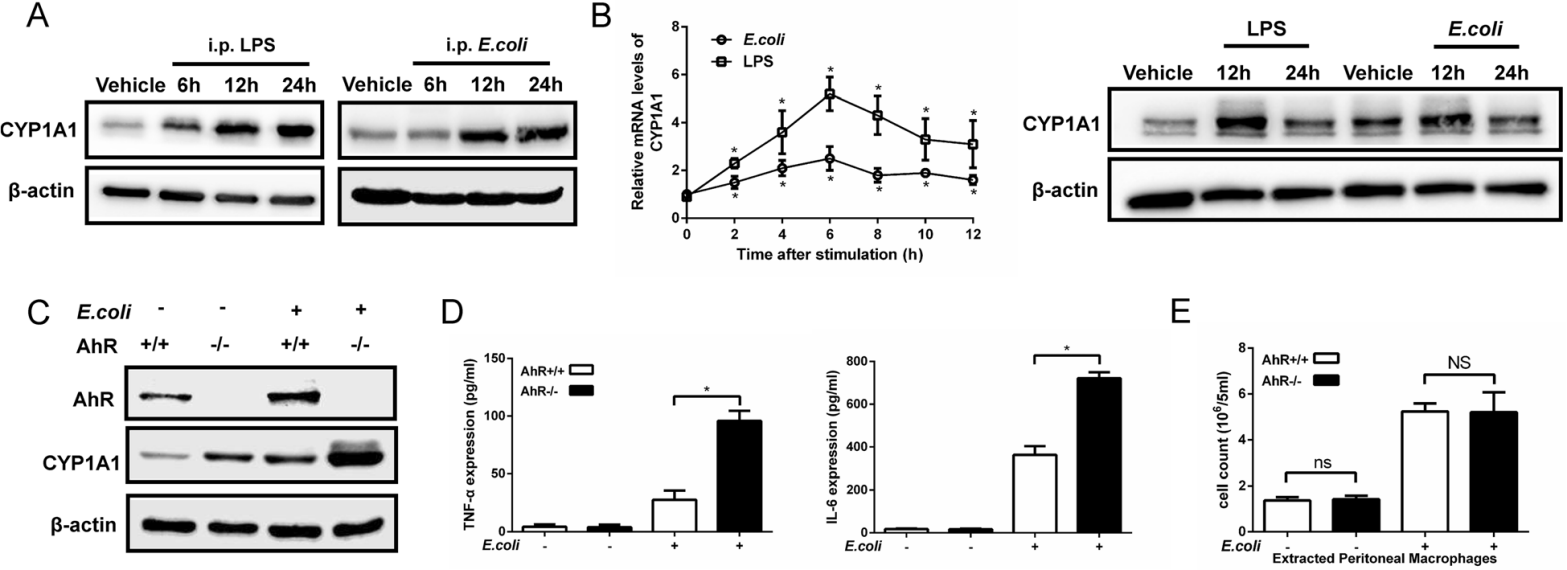
CYP1A1 is upregulated in PMs of septic mice. **a** Mice were intraperitoneally injected with vehicle (isopyknic PBS), LPS (20 mg/kg) or *E. coli* (1.2 × 10^11^ CFUs/kg, CFUs, colony forming units). PMs were extracted at the indicated times and subjected to western blotting analysis of CYP1A1 protein levels. **b** PMs isolated from WT mice were treated with vehicle, LPS (10 μg/ml) or heat-killed *E. coli* (MOIs = 10, MOIs, multiplicity of infections) for the indicated times. CYP1A1 mRNA expression was quantified by qRT-PCR. Expression levels of CYP1A1 protein were detected by western blotting. **c**-**e** AhR^−/−^ and WT mice were intraperitoneally injected with vehicle or *E.coli*. After 12 h treatment, PMs and PLFs were extracted and subjected to analysis of AhR and CYP1A1 protein expression levels (**a**), pro-inflammatory cytokines expression levels (**b**) and PMs count (**c**). Data are mean ± SEM of three independent experiments. Results were compared by one-way ANOVA. *p < 0.05. NS, no statistical difference

**Fig. 2:**
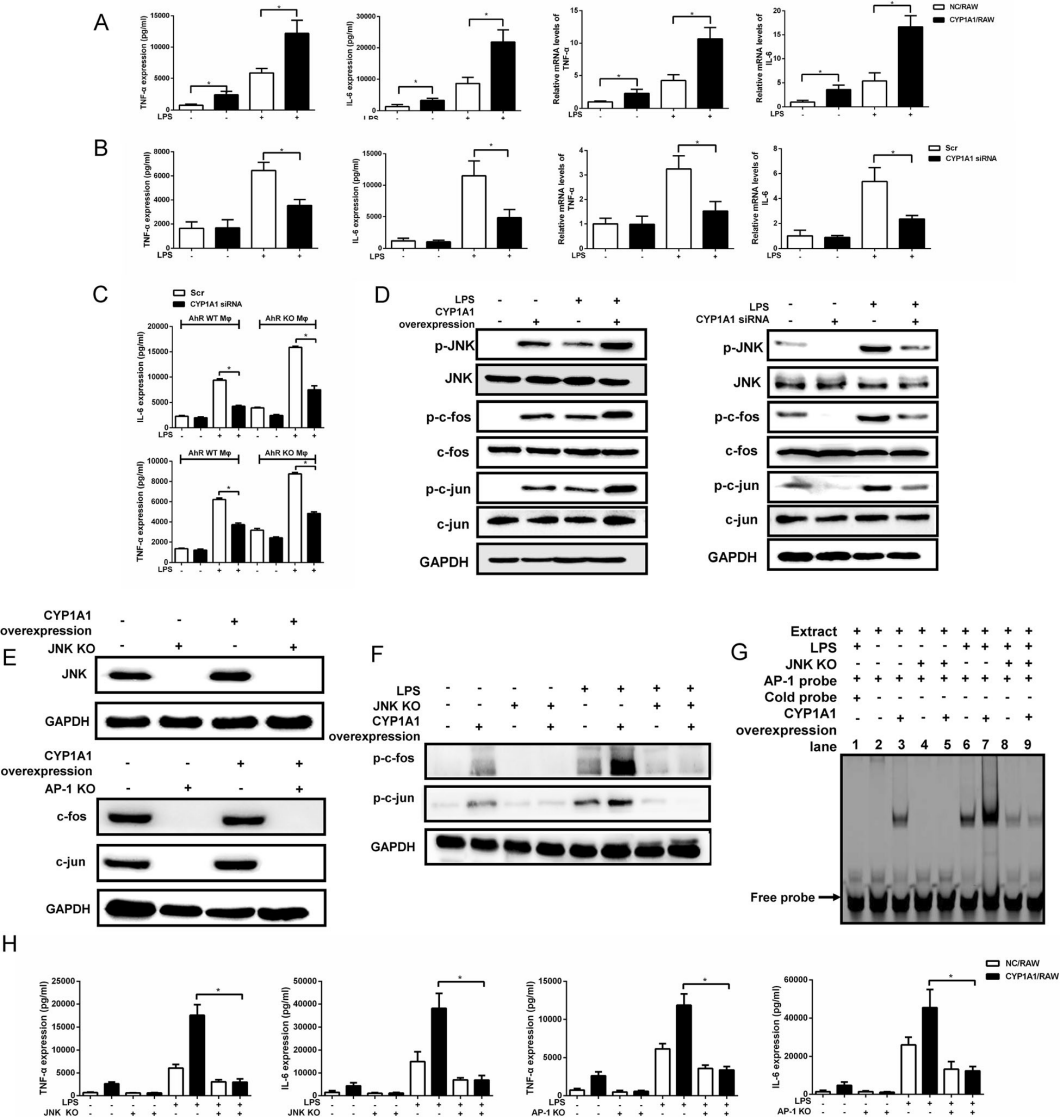
Elevation of 12(S)-HETE in LPS-stimulated CYP1A1/RAW cells is CYP1A1 hydroxylase-dependent rather than 12 lipoxygenase-dependent. **a**, **b** CYP1A1/RAW and NC/RAW were stimulated with vehicle or LPS (10 μg/ml) for 2 h for qRT-PCR or 12 h for western blotting. **a** 12-LOX mRNA levels were detected using qRT-PCR. 12-LOX protein levels were assessed by western blotting. **b**, **c** CYP1A1/RAW and NC/RAW were pre-treated with the selective 12-LOX inhibitor ML355 (10 μM) for 2 h and then stimulated with LPS for 12 h. **b** Supernatants were collected for CYP1A1 AHH activity measurement using a standard AHH activity assay protocol. **c** Alternatively, TNF-α, IL-6 and 12(S)-HETE levels were detected in supernatants using ELISA. **d** Schematic of CYP1A1 cDNA nucleotide sequence containing two mutant positions that impair CYP1A1 AHH activity. **e**, **h** CY1A1/RAW, NC/RAW and CYP1A1 mutant/RAW were treated with LPS for 12 h. **e** CYP1A1 protein levels were measured from cell lysate by western blotting. CYP1A1 AHH activity was measured in supernatants using a standard AHH activity assay. **f**, **g** CY1A1/RAW, NC/RAW and CYP1A1 mutant/RAW were treated with LPS for 2 h. **f** Treated cells were lysed and subjected to western blotting analysis. **g** The nuclear-extract proteins of treated cells were incubated with AP-1-binding site probe and binding activity measured by EMSA. **h** Supernatants were collected for analysis of TNF-α, IL-6, 12(S)-HETE and 14, 15-EET levels using ELISA. Data shown as mean ± SEM of three independent experiments. Results were compared by one-way ANOVA. *p < 0.05. NS, no statistical difference

## Supplementary information

**Additional file 1: Figure S4.** Validation of the NF-κB signalling pathway and different MAPK signalling pathways in LPS-stimulated CYP1A1/RAW and NC/RAW.
