# Supplementary material for: Cytochrome P450 1A1 enhances inflammatory responses and impedes phagocytosis of bacteria in macrophages during sepsis
Source: Cell Commun Signal. 2020 May 4;18:70. doi: 10.1186/s12964-020-0523-3 (PMC7199371; doi:10.1186/s12964-020-0523-3)
Supplement: Supplementary file 2 — Additional file 1: Figure S1. Confirmation of transfections. Figure S2. Relative expression levels of inflammatory factors in overactivated macrophages. Figure S3. The inhibitory effects of Rhapontigenin on LPS-induced TNF-α and IL-6 secretion in PMs. Figure S4. Validation of the NF-κB signalling pathway and different MAPK signalling pathways in LPS-stimulated CYP1A1/RAW and NC/RAW. Figure S5. The levels of 12(S)-HETE in PLFs from E.coli- and CLP-induced septic mice. Figure S6. Detection of lentivirus infection rate in PMs. Figure S7. The regulation of CYP1A1-JNK-AP-1 axis in septic mice. Figure S8. Platelet count in PLFs from CYP1A1-overexpressed macrophages transferred septic mice. Figure S9. CYP1A1 is involved in phagocytosis of bacteria in macrophages during sepsis. [file 12964_2020_523_MOESM2_ESM.zip › Supplemental Figure Legends.docx]

**Supplemental Figure legends**

**
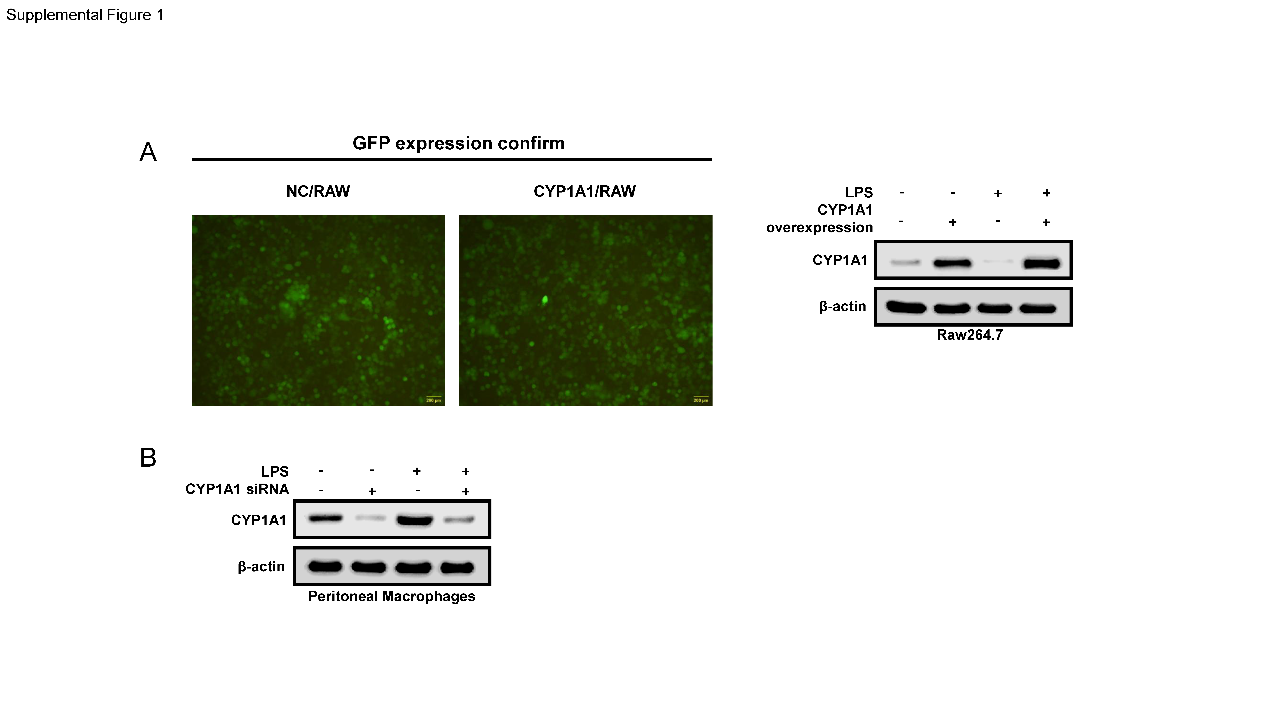
**

**Figure 1. Confirmation of transfections**

(A) RAW cells were infected with negative control-GFP, CYP1A1-overexpression-GFP lentivirus for 72 h and GFP expression levels were observed by fluorescence microscopy. Bar: 200 μm. CYP1A1 protein was measured in CYP1A1/RAW and NC/RAW following LPS (10 *μ*g/ml, 12 h) stimulation. (B) CYP1A1 protein levels were measured in mice PMs transfected with CYP1A1 siRNA or scramble siRNA prior to LPS stimulation.


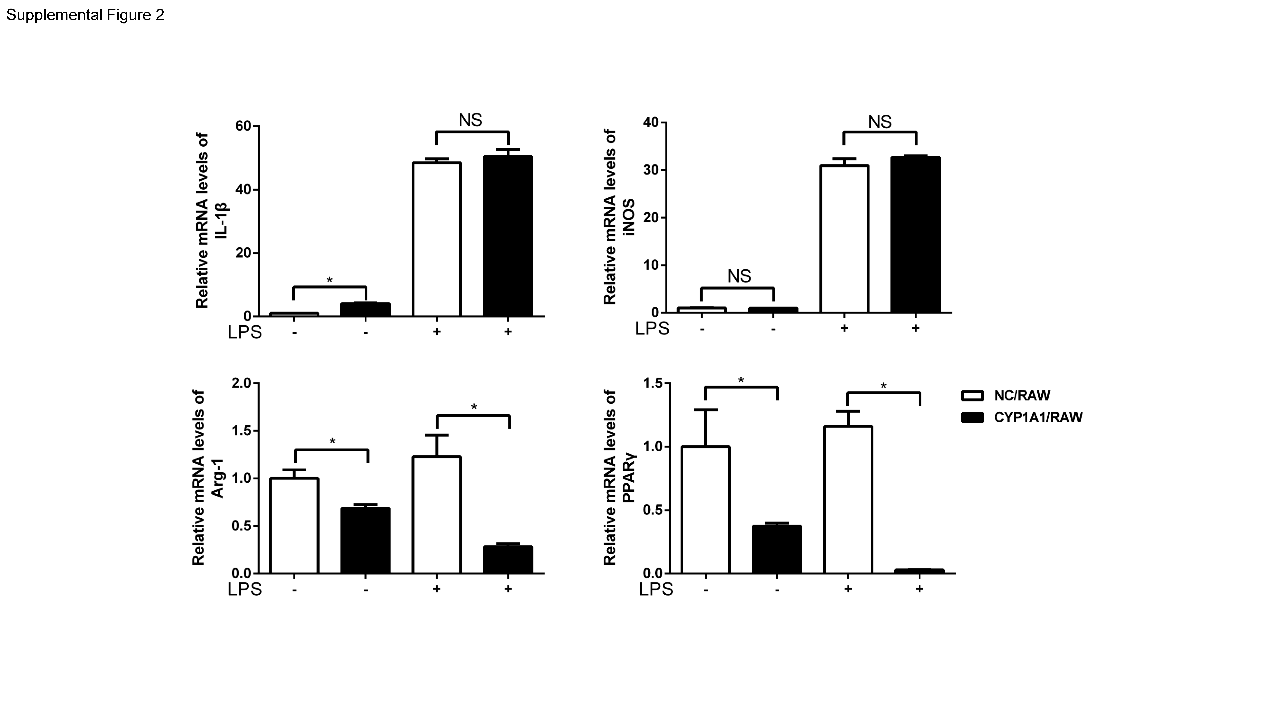


**Figure 2. Relative expression levels of inflammatory factors in overactivated macrophages**

CYP1A1/RAW and NC/RAW were treated with vehicle or LPS (10 *μ*g/ml) for 2 h. The mRNA expression levels of IL-1β, NOS2, Arg-1 and PPARγ were determined by qRT-PCR. Data are mean ± SEM of three independent experiments. Results were compared by one-way ANOVA. **p* < 0.05. NS, no statistical difference.


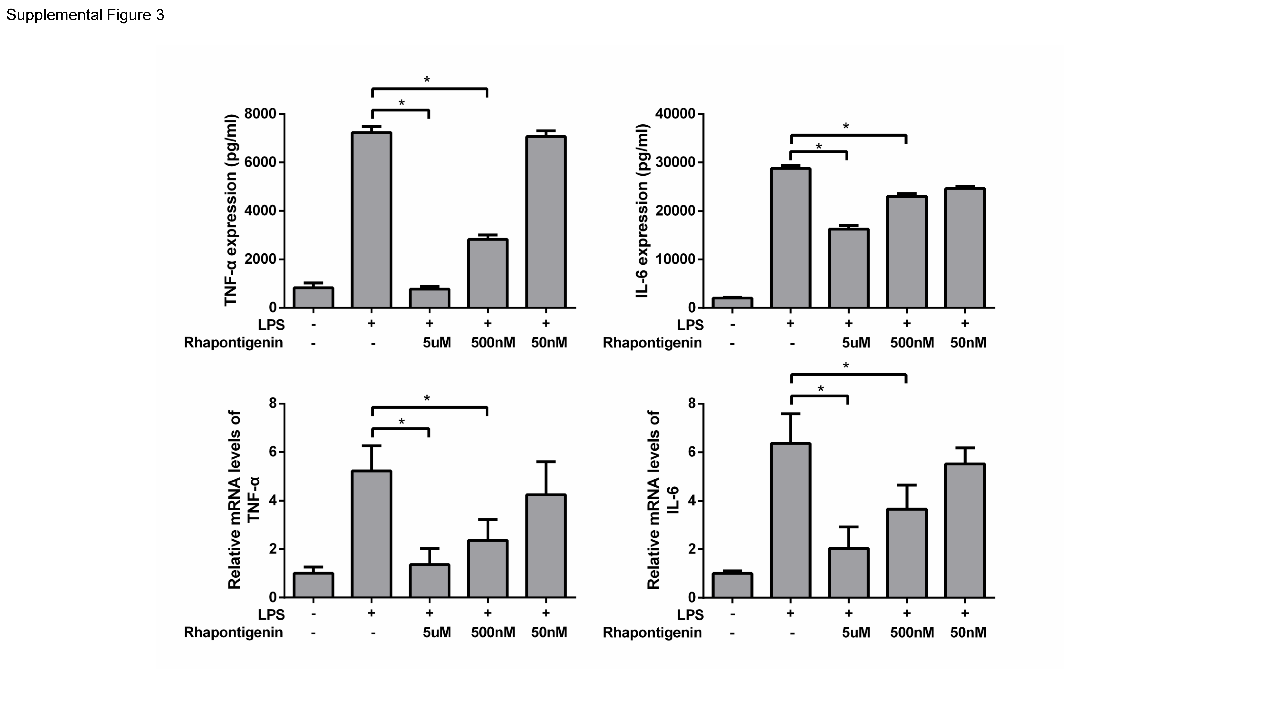


**Figure 3. The inhibitory effects of Rhapontigenin on LPS-induced TNF-α and IL-6 secretion in PMs**

PMs were pre-treated with the CYP1A1 inhibitor Rhapontigenin (5 *μ*M, 500 nM and 50 nM) for 2 h and then stimulated with vehicle or LPS (10 *μ*g/ml) for 12 h. TNF-α and IL-6 protein and mRNA levels were assessed by ELISA and qRT-PCR, respectively. Data are mean ± SEM of three independent experiments. Results were compared by one-way ANOVA. **p*< 0.05.


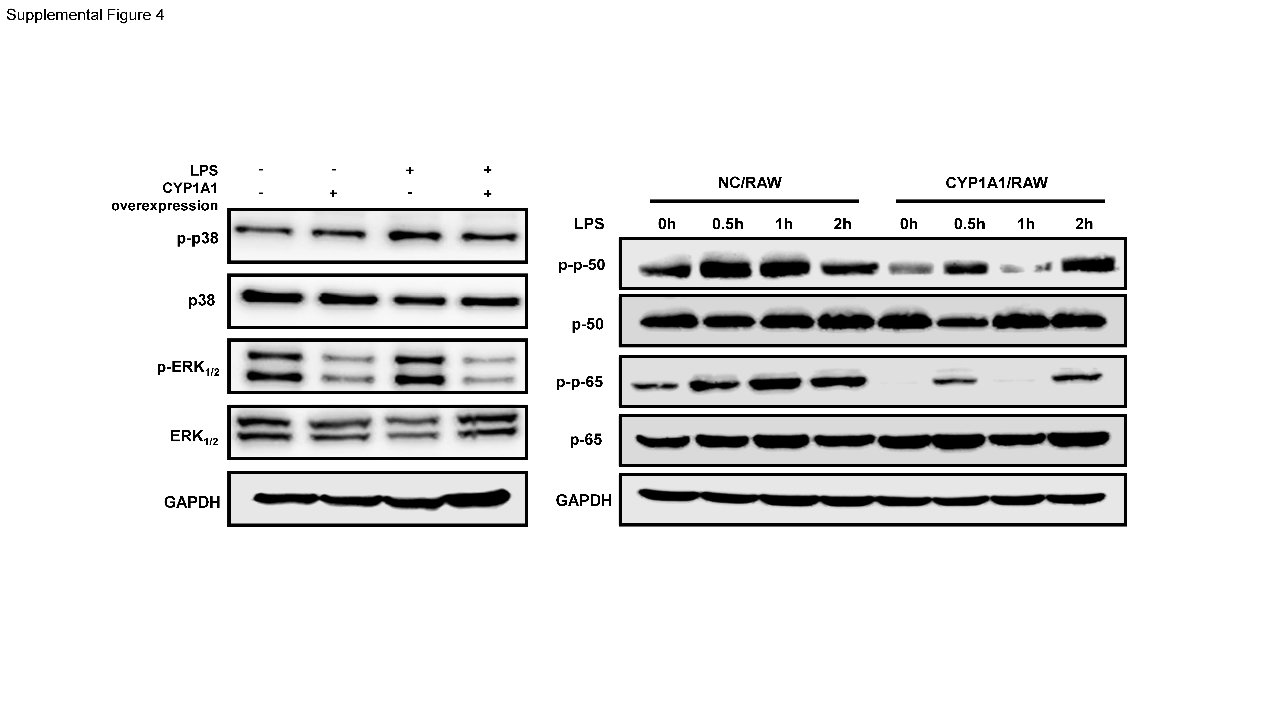


**Figure 4. Validation of the NF**-**κB signalling pathway and different MAPK signalling pathways in LPS-stimulated CYP1A1/RAW and NC/RAW**

CYP1A1/RAW and NC/RAW were treated with vehicle or LPS (10 *μ*g/ml) for 2 h. The cell lysates were analysed for p50, p65, p38 and ERK_1/2_ phosphorylation by western blot, respectively.





**Figure 5. The levels of 12(S)-HETE in PLFs from *E.coli*- and CLP-induced septic mice**

Mice were intraperitoneally injected with *E. coli* (1.2 × 10^11^ CFUs/kg) or received CLP surgery. PLFs were extracted at the indicated times for analysis of 12(S)-HETE levels using ELISA (n = 6). Results were compared by one-way ANOVA. Data are shown as mean ± SEM of three independent experiments. **p*< 0.05.


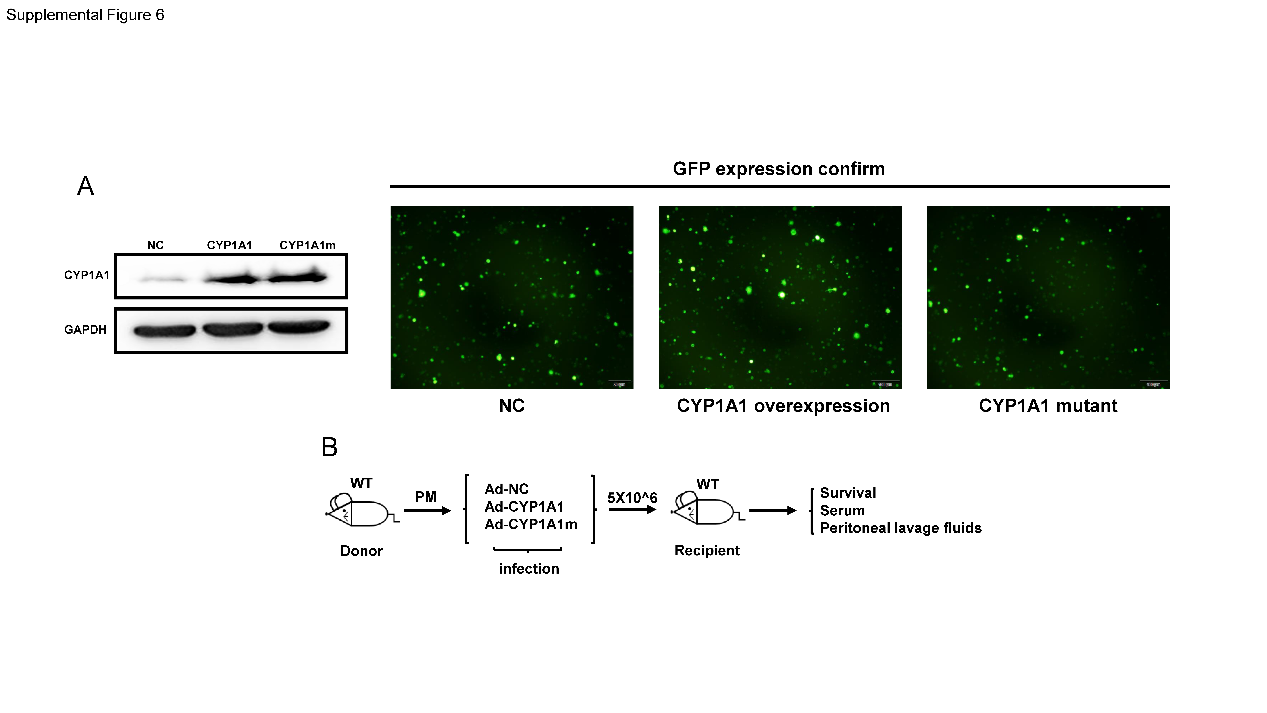


**Figure 6. Detection of lentivirus infection rate in PMs**

(A) Ad-CYP1A1, Ad-NC and Ad-CYP1A1m PMs were lysed for analysis of CYP1A1 protein levels. GFP expression levels were also observed by fluorescence microscopy. Bar: 100 μm. (B) PMs transfected with Ad-NC, Ad-CYP1A1 or Ad-CYP1A1m were injected intraperitoneally into WT mice 2 days before *E. coli* or CLP impact.


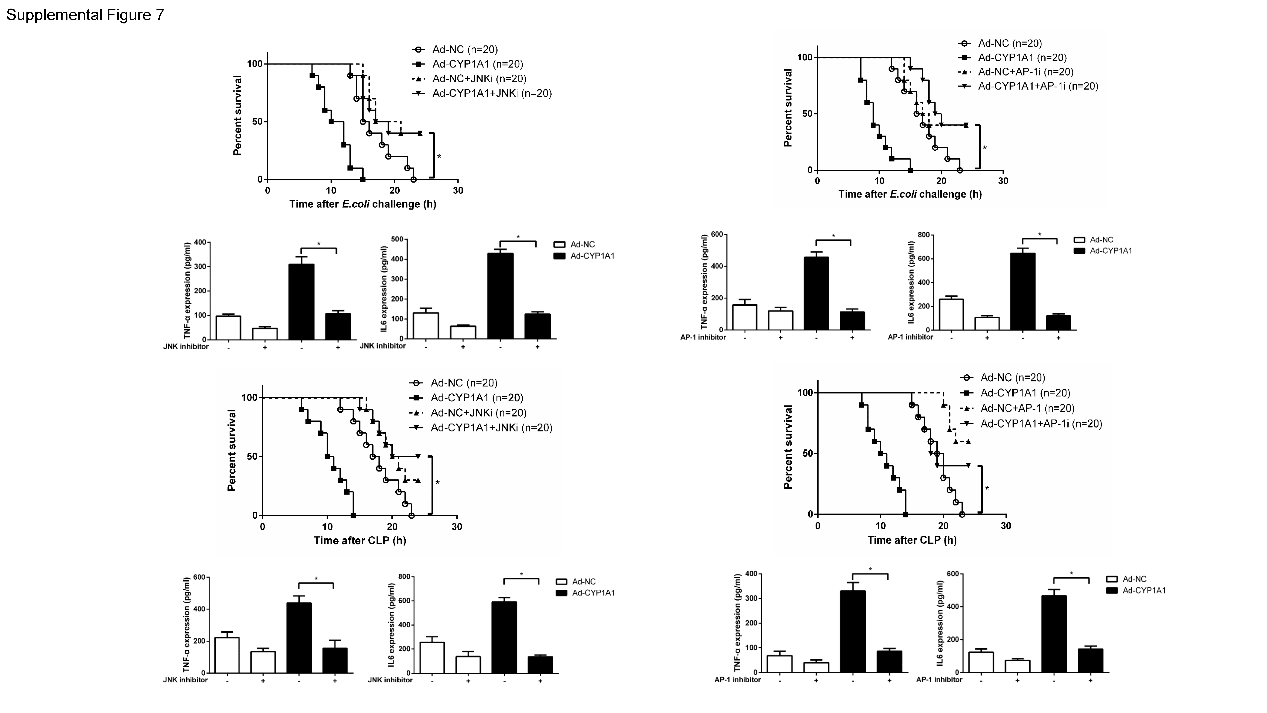


**Figure 7. The regulation of CYP1A1-JNK-AP-1 axis in septic mice**

PMs transfected with Ad-NC or Ad-CYP1A1 were injected intraperitoneally into WT mice. After 2 days, mice were treated with vehicle, JNK inhibitor (30 mg/kg), or AP-1 inhibitor (20 mg/kg) 2h before *E. coli* (1.2 × 10^11^ CFUs/kg) or CLP challenge. In survival experiments, JNK and AP-1 inhibitors were re-injected at 6, 12 and 24 h, respectively, following *E. coli* or CLP impact. Survival rates were monitored for 2 days after *E. coli* or CLP impact and presented as Kaplan-Meier survival curves. Results were compared by log-rank test (n = 20). PLFs were collected from each group 12 h after *E. coli* or CLP impact and TNF-α and IL-6 protein levels were detected by ELISA (n = 6). Results were compared by one-way ANOVA. Data shown as mean ± SEM. **p* < 0.05.


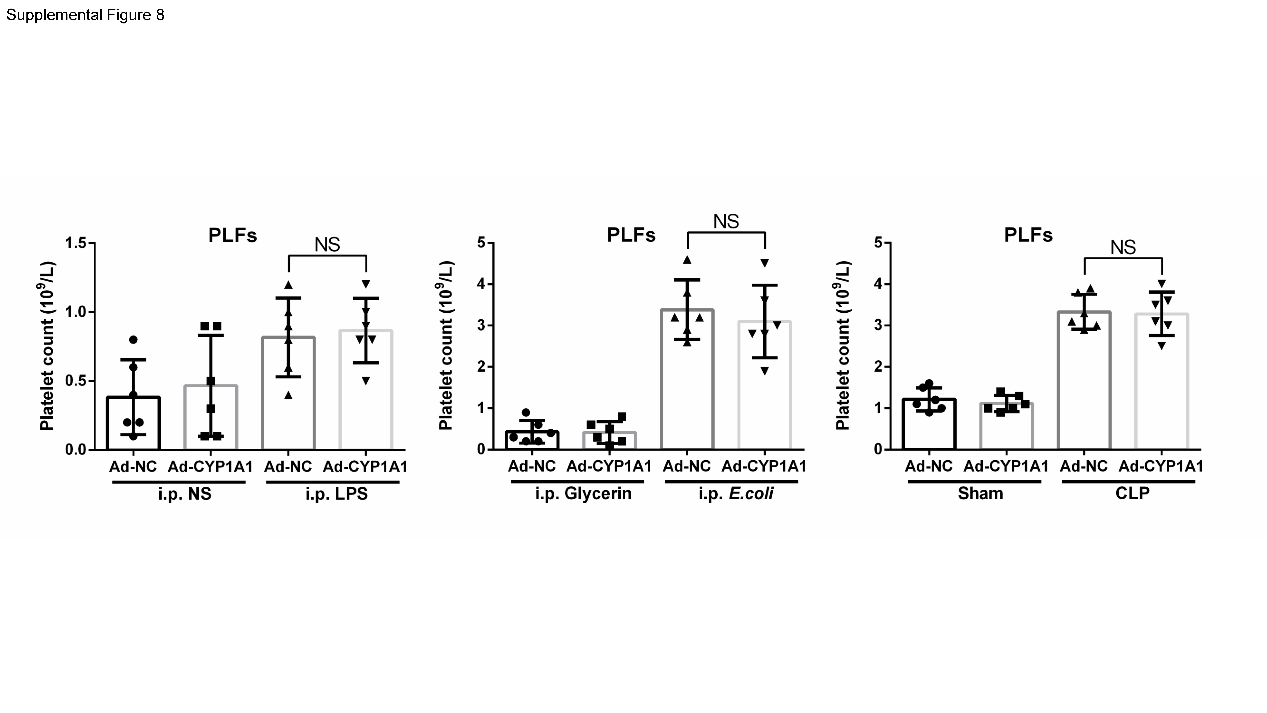


**Figure 8. Platelet count in PLFs from CYP1A1-overexpressed macrophages transferred septic mice**

PMs transfected with Ad-NC or Ad-CYP1A1 were injected intraperitoneally into WT mice 2 days before LPS (30 mg/kg), *E. coli* (1.2 × 10^11^ CFUs/kg) or CLP impact. PLFs were extracted for platelet counts after 12 h challenge. Group means were compared by Student’s *t* test (n=6). Data are shown as mean ± SEM of three independent experiments. **p* < 0.05. NS, no statistical difference.


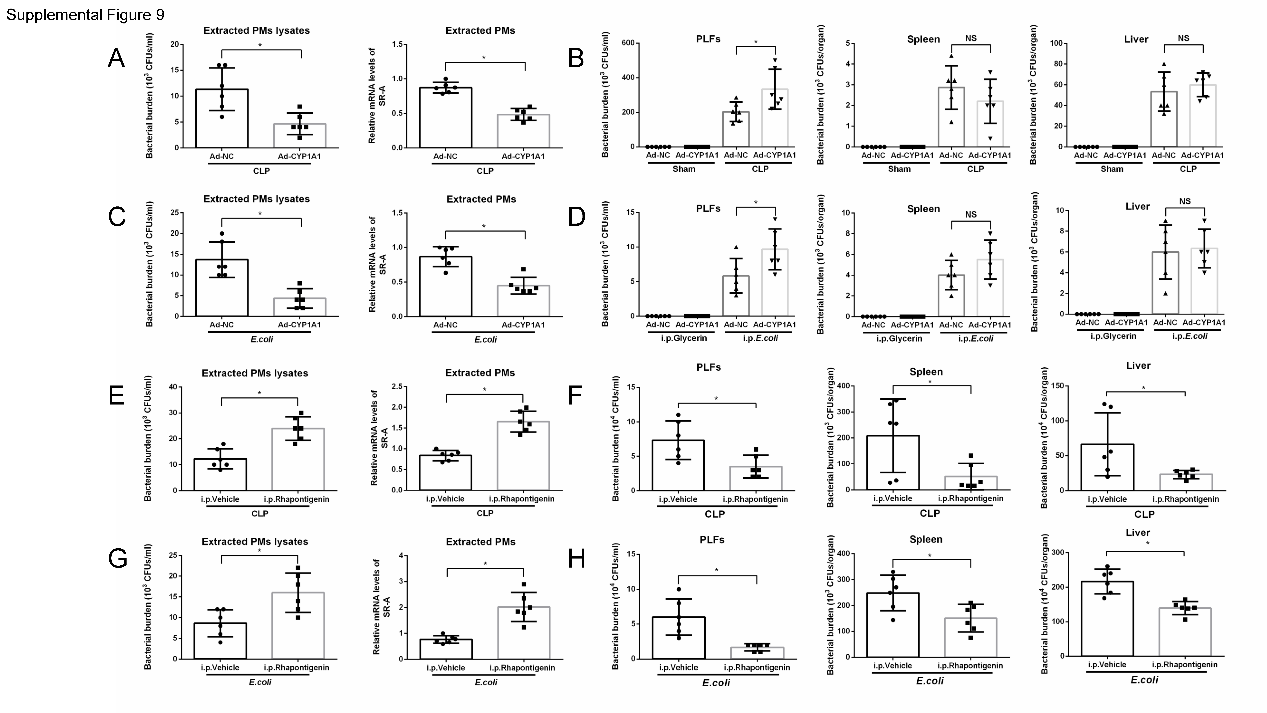


**Figure 9. CYP1A1 is involved in phagocytosis of bacteria in macrophages during sepsis**

(A-D) PMs transfected with Ad-NC or Ad-CYP1A1 were injected intraperitoneally into WT mice 30 minutes before *E. coli* (1.2 × 10^11^ CFUs/kg) impact or at the same time with CLP impact. (E-H) WT mice were pre-treated with Rhapontigenin (10 mg/kg) for 1 h before *E. coli* or CLP impact. (A, C, E, G) PMs were extracted after 40 minutes challenge and subjected for intracellular bacteria count and SR-A mRNA level measurement. (B, D, F, H) PLFs, lysates of spleen and liver were obtained for survival bacterial colonies counts after 12 h challenge. Group means were compared by Student’s *t* test (n=6).**p* < 0.05. NS, no statistical difference.
