# Supplementary figures and images for: Cytochrome P450 1A1 enhances inflammatory responses and impedes phagocytosis of bacteria in macrophages during sepsis
Source: Cell Commun Signal. 2020 May 4;18:70. doi: 10.1186/s12964-020-0523-3 (PMC7199371; doi:10.1186/s12964-020-0523-3)

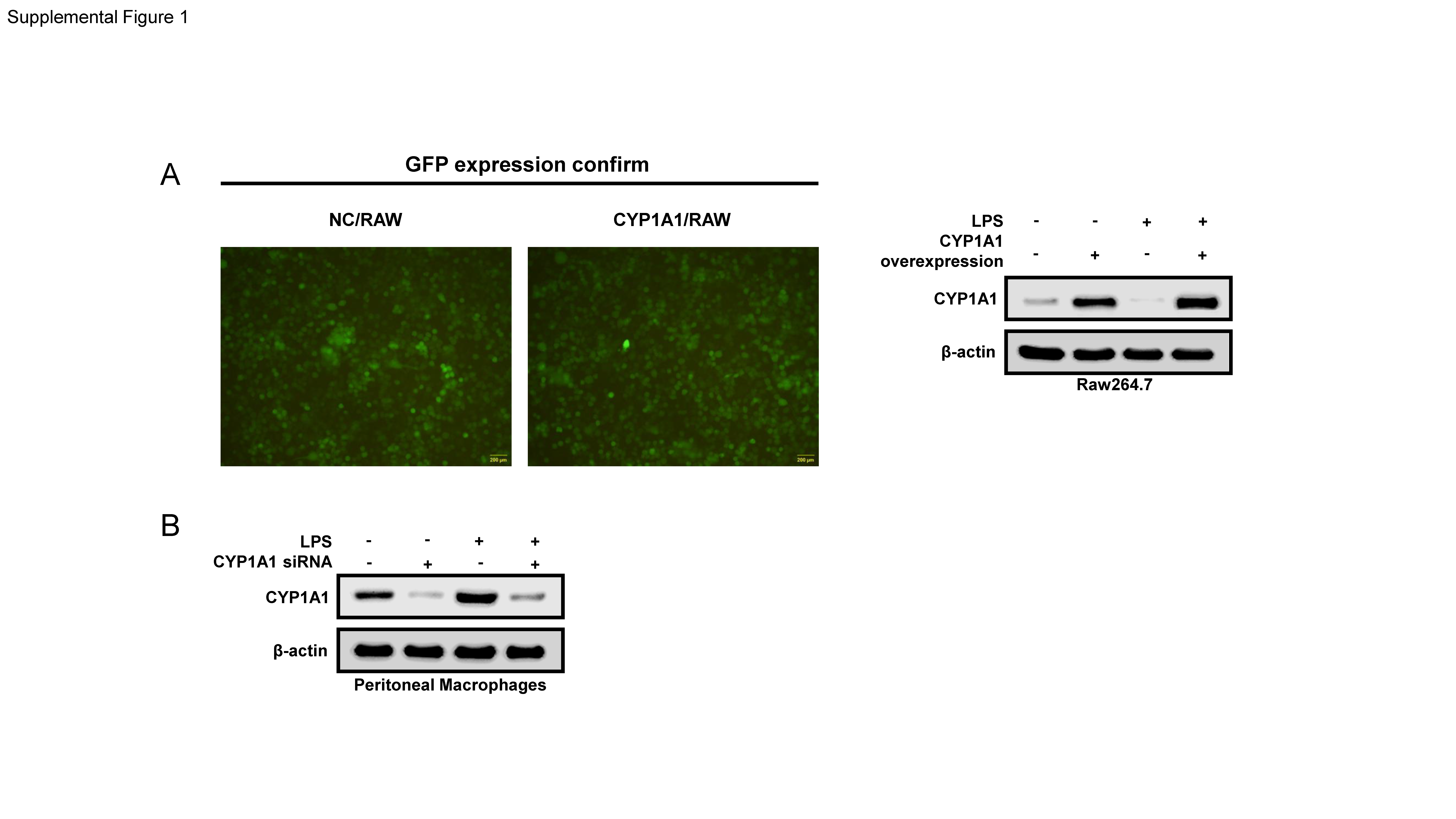

Supplement: Supplementary file 2 — Additional file 1: Figure S1. Confirmation of transfections. Figure S2. Relative expression levels of inflammatory factors in overactivated macrophages. Figure S3. The inhibitory effects of Rhapontigenin on LPS-induced TNF-α and IL-6 secretion in PMs. Figure S4. Validation of the NF-κB signalling pathway and different MAPK signalling pathways in LPS-stimulated CYP1A1/RAW and NC/RAW. Figure S5. The levels of 12(S)-HETE in PLFs from E.coli- and CLP-induced septic mice. Figure S6. Detection of lentivirus infection rate in PMs. Figure S7. The regulation of CYP1A1-JNK-AP-1 axis in septic mice. Figure S8. Platelet count in PLFs from CYP1A1-overexpressed macrophages transferred septic mice. Figure S9. CYP1A1 is involved in phagocytosis of bacteria in macrophages during sepsis. [file 12964_2020_523_MOESM2_ESM.zip › Supplemental Figure 1.tif]

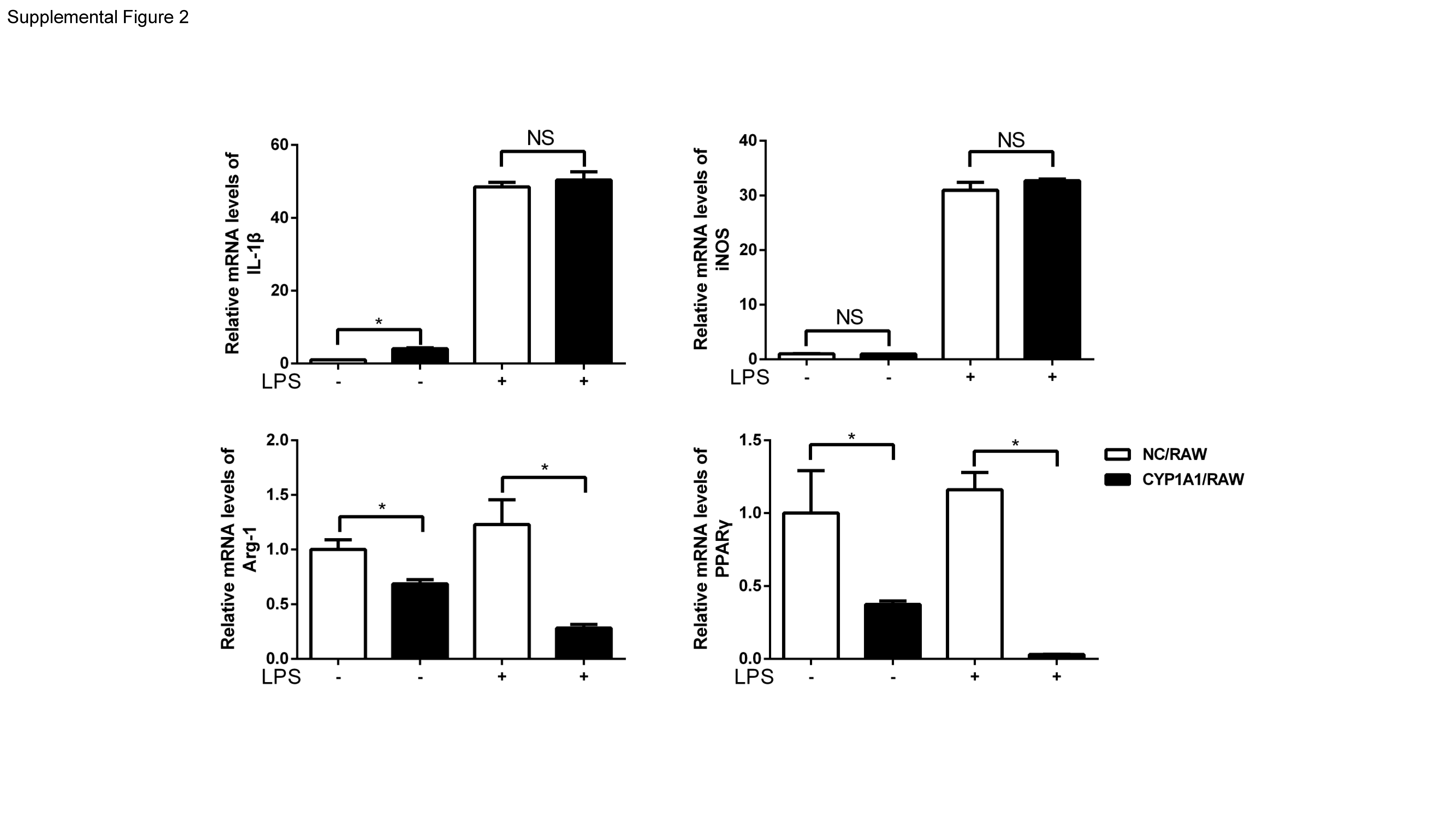

Supplement: Supplementary file 2 — Additional file 1: Figure S1. Confirmation of transfections. Figure S2. Relative expression levels of inflammatory factors in overactivated macrophages. Figure S3. The inhibitory effects of Rhapontigenin on LPS-induced TNF-α and IL-6 secretion in PMs. Figure S4. Validation of the NF-κB signalling pathway and different MAPK signalling pathways in LPS-stimulated CYP1A1/RAW and NC/RAW. Figure S5. The levels of 12(S)-HETE in PLFs from E.coli- and CLP-induced septic mice. Figure S6. Detection of lentivirus infection rate in PMs. Figure S7. The regulation of CYP1A1-JNK-AP-1 axis in septic mice. Figure S8. Platelet count in PLFs from CYP1A1-overexpressed macrophages transferred septic mice. Figure S9. CYP1A1 is involved in phagocytosis of bacteria in macrophages during sepsis. [file 12964_2020_523_MOESM2_ESM.zip › Supplemental Figure 2.tif]

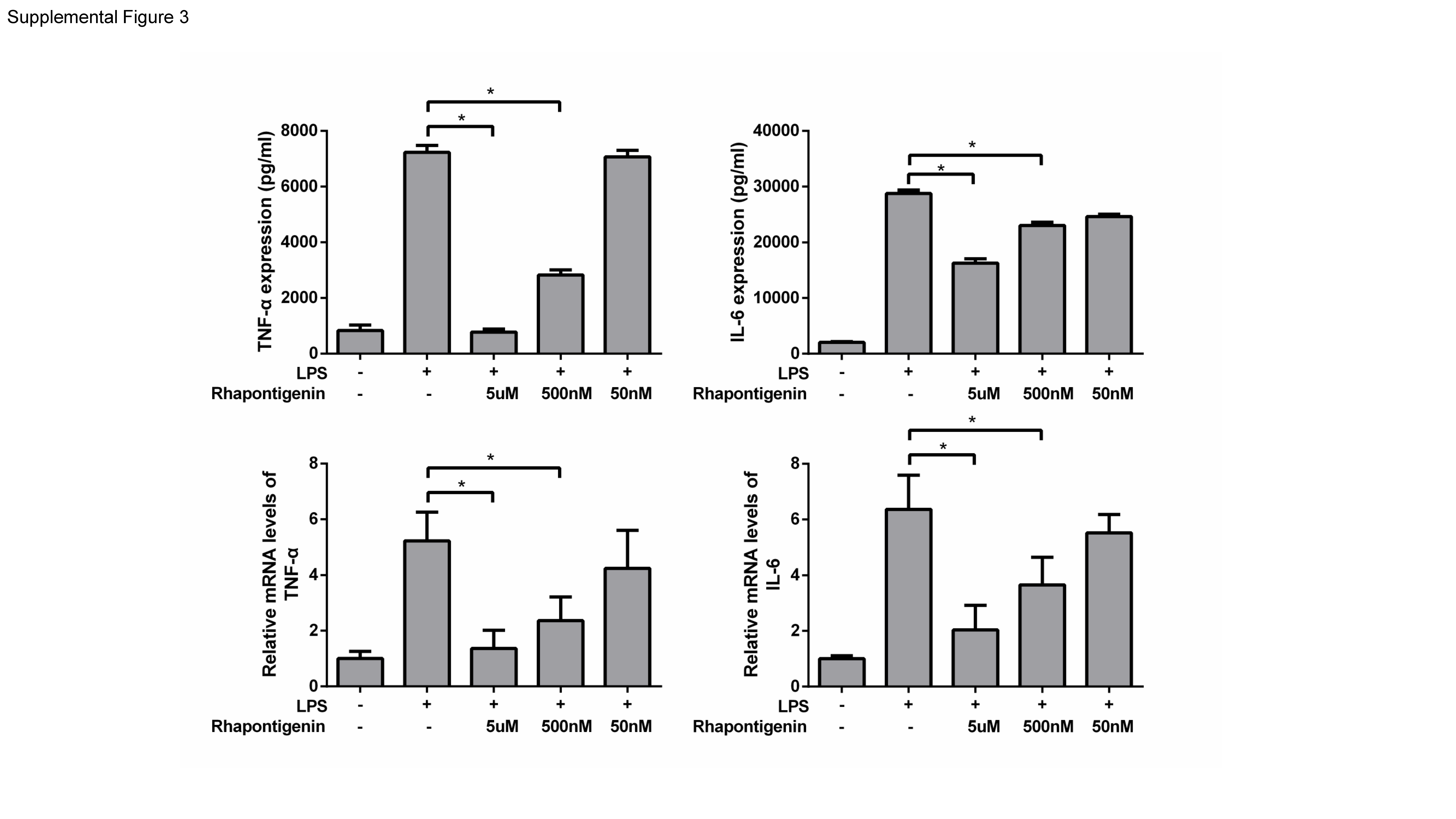

Supplement: Supplementary file 2 — Additional file 1: Figure S1. Confirmation of transfections. Figure S2. Relative expression levels of inflammatory factors in overactivated macrophages. Figure S3. The inhibitory effects of Rhapontigenin on LPS-induced TNF-α and IL-6 secretion in PMs. Figure S4. Validation of the NF-κB signalling pathway and different MAPK signalling pathways in LPS-stimulated CYP1A1/RAW and NC/RAW. Figure S5. The levels of 12(S)-HETE in PLFs from E.coli- and CLP-induced septic mice. Figure S6. Detection of lentivirus infection rate in PMs. Figure S7. The regulation of CYP1A1-JNK-AP-1 axis in septic mice. Figure S8. Platelet count in PLFs from CYP1A1-overexpressed macrophages transferred septic mice. Figure S9. CYP1A1 is involved in phagocytosis of bacteria in macrophages during sepsis. [file 12964_2020_523_MOESM2_ESM.zip › Supplemental Figure 3.tif]

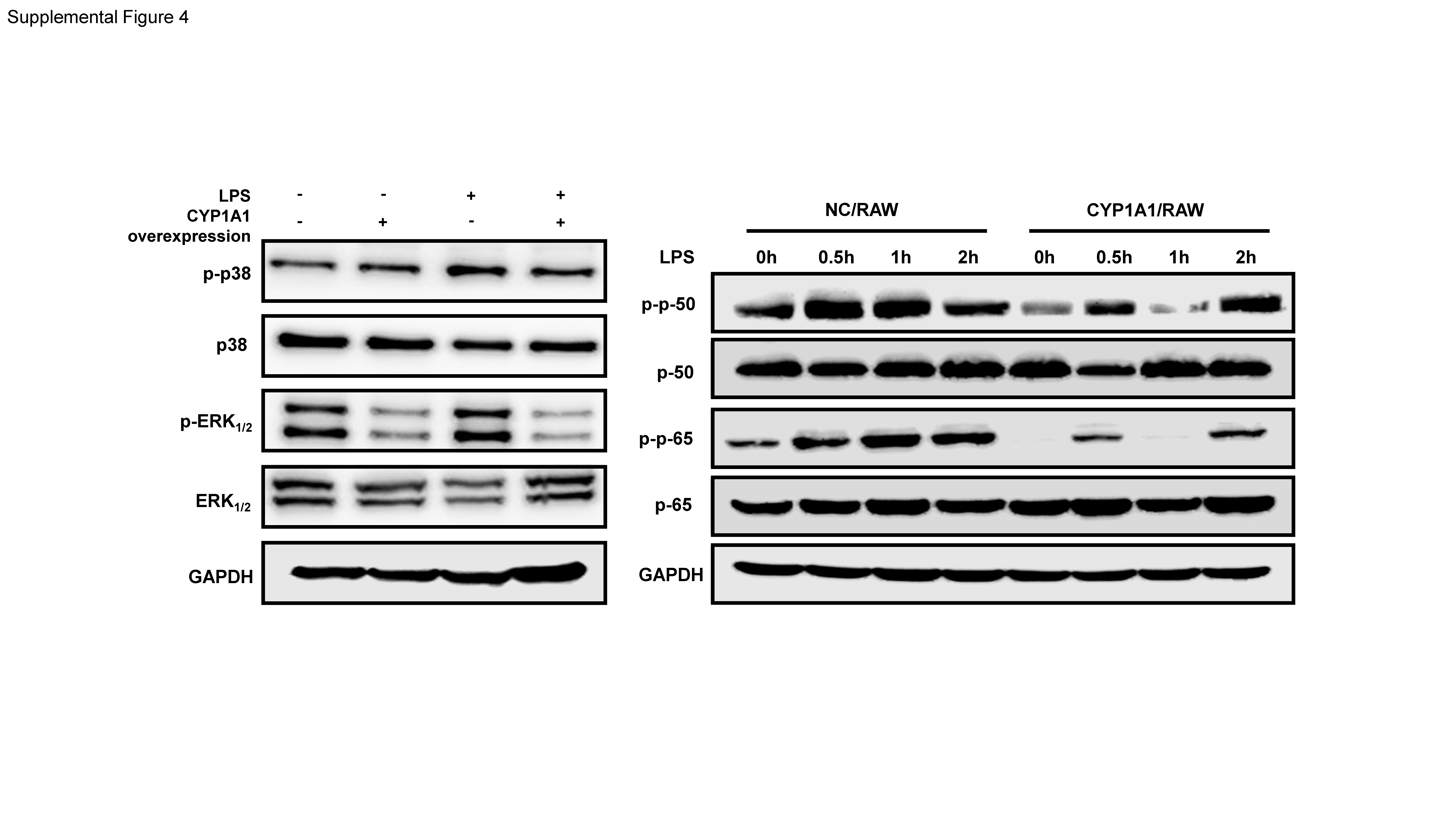

Supplement: Supplementary file 2 — Additional file 1: Figure S1. Confirmation of transfections. Figure S2. Relative expression levels of inflammatory factors in overactivated macrophages. Figure S3. The inhibitory effects of Rhapontigenin on LPS-induced TNF-α and IL-6 secretion in PMs. Figure S4. Validation of the NF-κB signalling pathway and different MAPK signalling pathways in LPS-stimulated CYP1A1/RAW and NC/RAW. Figure S5. The levels of 12(S)-HETE in PLFs from E.coli- and CLP-induced septic mice. Figure S6. Detection of lentivirus infection rate in PMs. Figure S7. The regulation of CYP1A1-JNK-AP-1 axis in septic mice. Figure S8. Platelet count in PLFs from CYP1A1-overexpressed macrophages transferred septic mice. Figure S9. CYP1A1 is involved in phagocytosis of bacteria in macrophages during sepsis. [file 12964_2020_523_MOESM2_ESM.zip › Supplemental Figure 4.tif]

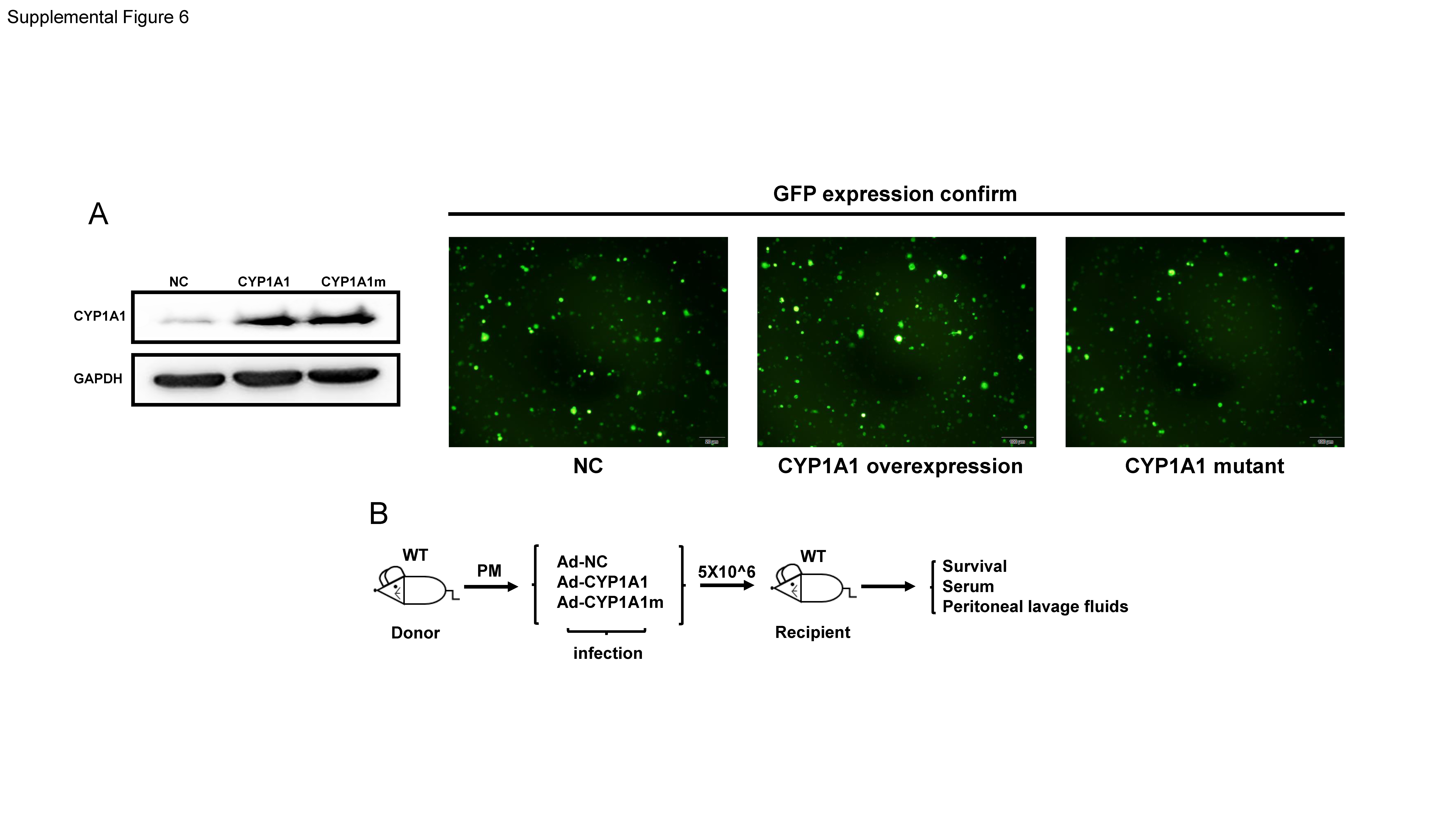

Supplement: Supplementary file 2 — Additional file 1: Figure S1. Confirmation of transfections. Figure S2. Relative expression levels of inflammatory factors in overactivated macrophages. Figure S3. The inhibitory effects of Rhapontigenin on LPS-induced TNF-α and IL-6 secretion in PMs. Figure S4. Validation of the NF-κB signalling pathway and different MAPK signalling pathways in LPS-stimulated CYP1A1/RAW and NC/RAW. Figure S5. The levels of 12(S)-HETE in PLFs from E.coli- and CLP-induced septic mice. Figure S6. Detection of lentivirus infection rate in PMs. Figure S7. The regulation of CYP1A1-JNK-AP-1 axis in septic mice. Figure S8. Platelet count in PLFs from CYP1A1-overexpressed macrophages transferred septic mice. Figure S9. CYP1A1 is involved in phagocytosis of bacteria in macrophages during sepsis. [file 12964_2020_523_MOESM2_ESM.zip › Supplemental Figure 6.tif]

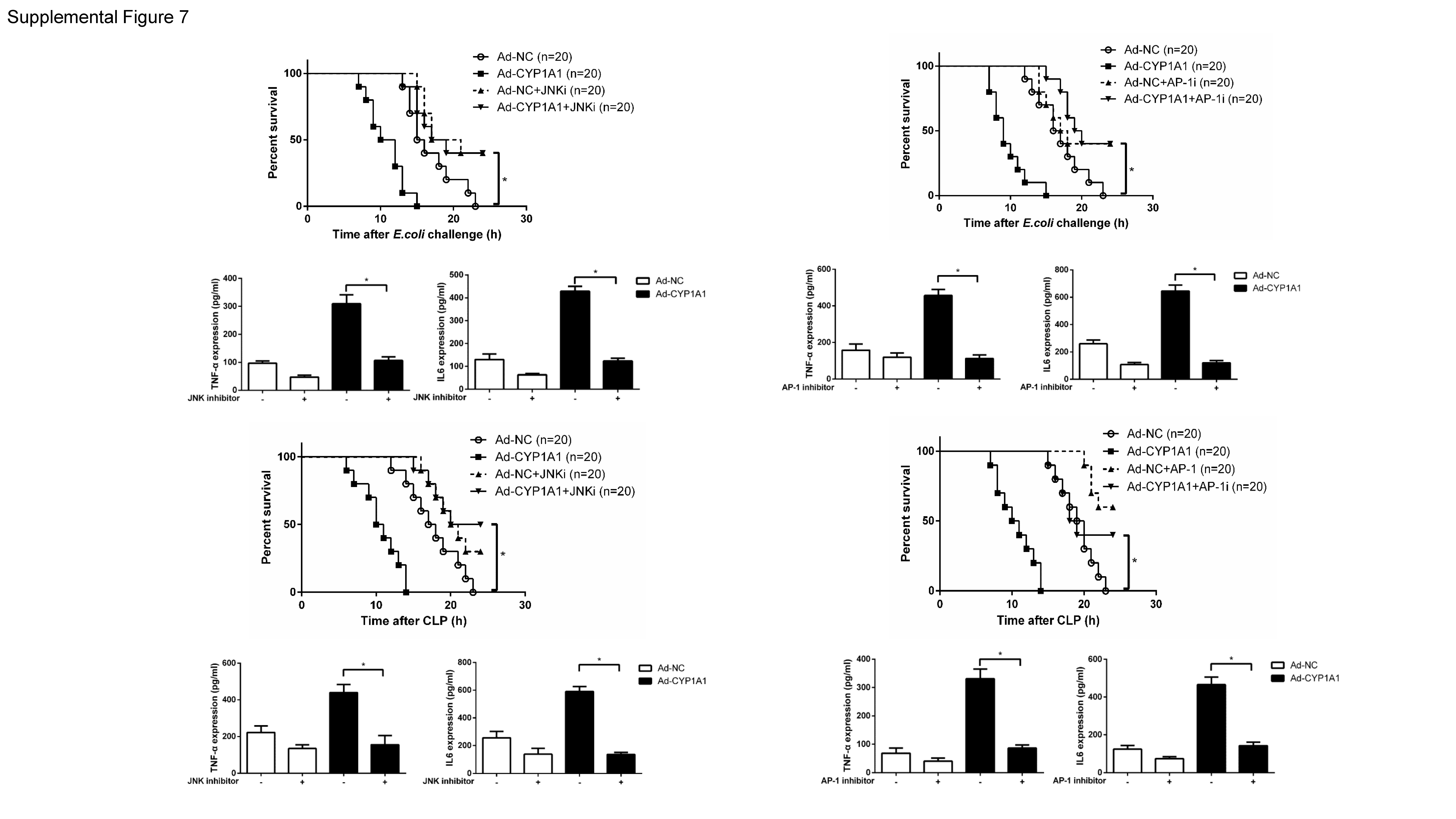

Supplement: Supplementary file 2 — Additional file 1: Figure S1. Confirmation of transfections. Figure S2. Relative expression levels of inflammatory factors in overactivated macrophages. Figure S3. The inhibitory effects of Rhapontigenin on LPS-induced TNF-α and IL-6 secretion in PMs. Figure S4. Validation of the NF-κB signalling pathway and different MAPK signalling pathways in LPS-stimulated CYP1A1/RAW and NC/RAW. Figure S5. The levels of 12(S)-HETE in PLFs from E.coli- and CLP-induced septic mice. Figure S6. Detection of lentivirus infection rate in PMs. Figure S7. The regulation of CYP1A1-JNK-AP-1 axis in septic mice. Figure S8. Platelet count in PLFs from CYP1A1-overexpressed macrophages transferred septic mice. Figure S9. CYP1A1 is involved in phagocytosis of bacteria in macrophages during sepsis. [file 12964_2020_523_MOESM2_ESM.zip › Supplemental Figure 7.tif]

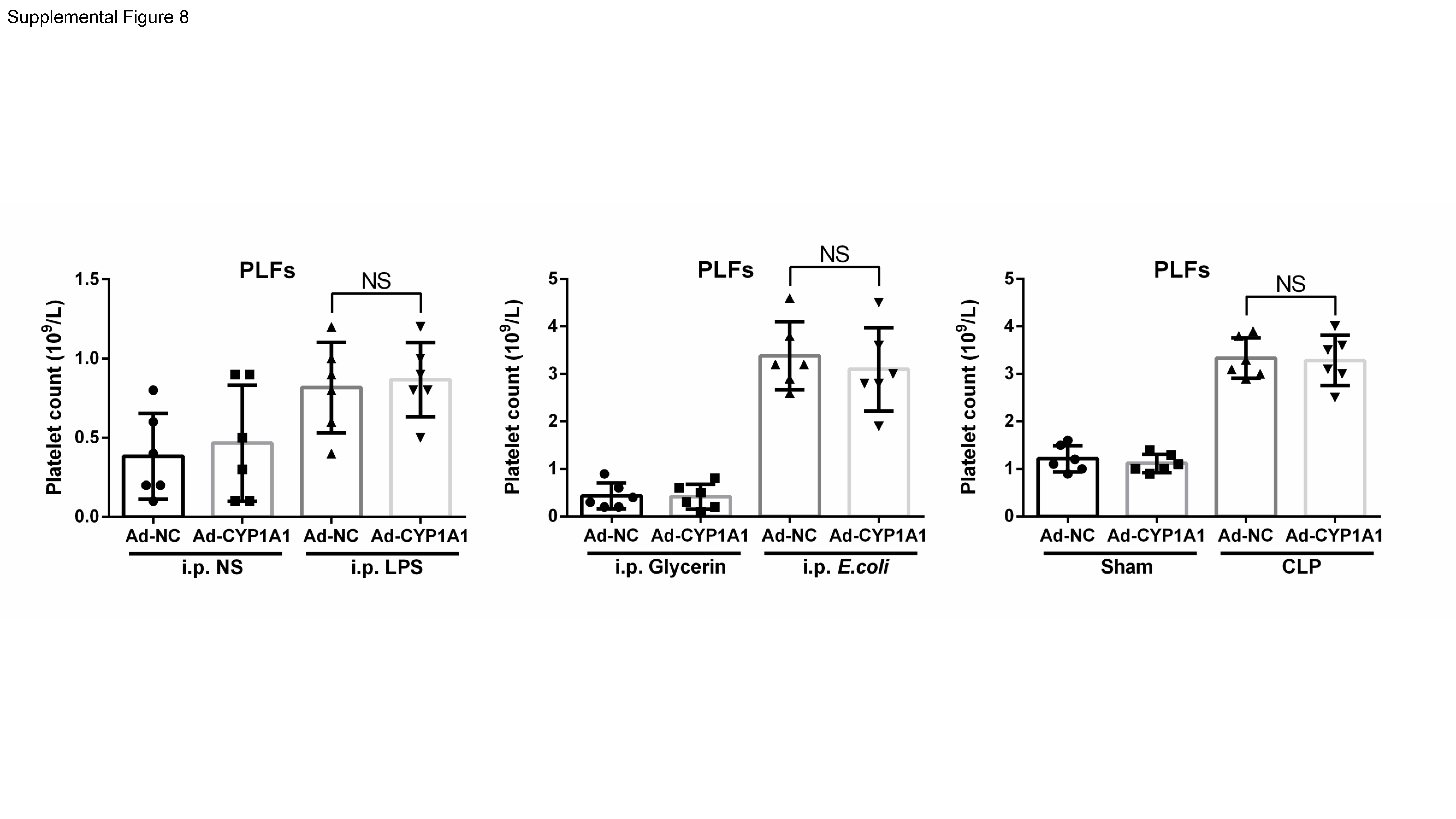

Supplement: Supplementary file 2 — Additional file 1: Figure S1. Confirmation of transfections. Figure S2. Relative expression levels of inflammatory factors in overactivated macrophages. Figure S3. The inhibitory effects of Rhapontigenin on LPS-induced TNF-α and IL-6 secretion in PMs. Figure S4. Validation of the NF-κB signalling pathway and different MAPK signalling pathways in LPS-stimulated CYP1A1/RAW and NC/RAW. Figure S5. The levels of 12(S)-HETE in PLFs from E.coli- and CLP-induced septic mice. Figure S6. Detection of lentivirus infection rate in PMs. Figure S7. The regulation of CYP1A1-JNK-AP-1 axis in septic mice. Figure S8. Platelet count in PLFs from CYP1A1-overexpressed macrophages transferred septic mice. Figure S9. CYP1A1 is involved in phagocytosis of bacteria in macrophages during sepsis. [file 12964_2020_523_MOESM2_ESM.zip › Supplemental Figure 8.tif]

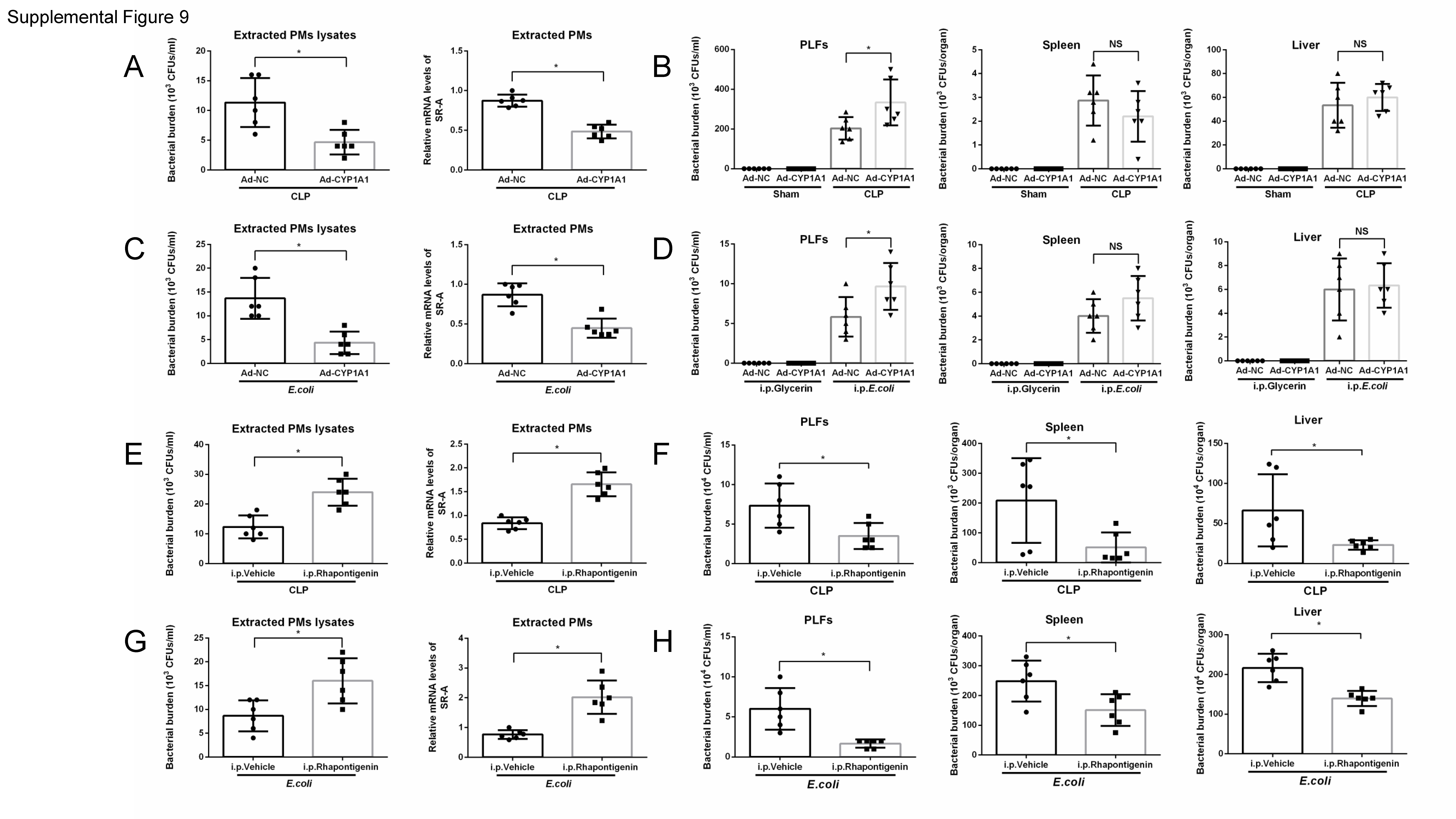

Supplement: Supplementary file 2 — Additional file 1: Figure S1. Confirmation of transfections. Figure S2. Relative expression levels of inflammatory factors in overactivated macrophages. Figure S3. The inhibitory effects of Rhapontigenin on LPS-induced TNF-α and IL-6 secretion in PMs. Figure S4. Validation of the NF-κB signalling pathway and different MAPK signalling pathways in LPS-stimulated CYP1A1/RAW and NC/RAW. Figure S5. The levels of 12(S)-HETE in PLFs from E.coli- and CLP-induced septic mice. Figure S6. Detection of lentivirus infection rate in PMs. Figure S7. The regulation of CYP1A1-JNK-AP-1 axis in septic mice. Figure S8. Platelet count in PLFs from CYP1A1-overexpressed macrophages transferred septic mice. Figure S9. CYP1A1 is involved in phagocytosis of bacteria in macrophages during sepsis. [file 12964_2020_523_MOESM2_ESM.zip › Supplemental Figure 9.tif]
