# Supplementary material for: Cytochrome P450 1A1 enhances inflammatory responses and impedes phagocytosis of bacteria in macrophages during sepsis
Source: Cell Commun Signal. 2020 May 4;18:70. doi: 10.1186/s12964-020-0523-3 (PMC7199371; doi:10.1186/s12964-020-0523-3)
Supplement: Supplementary file 3 — Additional file 2: Table S1. [file 12964_2020_523_MOESM3_ESM.docx]

| **Gene** | **Forward primer (5’-3’)** | **Reverse primer (5’-3’)** |
| --- | --- | --- |
| **Human** | | |
| **CYP1A1** | TCACAGACAGCCTGATTGAG | GATGGGTTGACCCATAGCTT |
| **TNF-α** | AACATCCAACCTTCCCAAACG | CCCTAAGCCCCCAATTCTCTT |
| **IL-6** | AATTCGGTACATCCCGACGG | GGTTGTGGGCTGCCAGTGCC |
| **GAPDH** | GTGAAGGTCGGAGTCAACG | GAGATGATGACCCTTTTGGC |
| **Mice** | | |
| **CYP1A1** | CAGGTAACGGAGGACAGGAA | GGCCACTTTGACCCTTACAA |
| **TNF-α** | GAGTCCGGGCAGGTCTACTTT | CAGGTCACTGTCCCAGCATCT |
| **IL-6** | ACCACGGCCTTCCCTACTTC | CTCATTTCCACGATTTCCCAG |
| **12-LOX** | TTCCAAGAGCCGCTTTCCATA | TCTATCACTAGCCCAAAACATC |
| **Arg-1** | AGAGCTGACAGCAACCCTGT | GGATCCAGAAGGTGATGGAA |
| **IL-β** | GCAACTGTTCCTGAAACTAACT | ATCCTTTTGGGGTCCGTCAACT |
| **iNOS** | ACCACTCGTACTTGGGATGT | CACCTTGGACTTCACCCAGT |
| **β-actin** | AGCCATGTACGTAGCCATCC | CTCTCAGCTGTGGTGGTGAA |
| **PPARγ** | CGCGGGAATTCGGTGAAACTCTGGGGAGATTC | CGCGGGATTCGTTGACACAGAGATGCCATTC |
| **SR-A** | CTTTACCAGCAATGACAAAAGAGA | ATTTCACGGATTCTGAACTGC |

Supplemental table 1
