# Supplementary material for: Cytochrome P450 1A1 enhances inflammatory responses and impedes phagocytosis of bacteria in macrophages during sepsis
Source: Cell Commun Signal. 2020 May 4;18:70. doi: 10.1186/s12964-020-0523-3 (PMC7199371; doi:10.1186/s12964-020-0523-3)
Supplement: Supplementary file 4 — Additional file 3: Table S2. [file 12964_2020_523_MOESM4_ESM.docx]

|  | Septic patients (n=30) | Healthy controls (n=30) | P value |
| --- | --- | --- | --- |
| Age (yr) | 51.63±15.96 | 46.50±14.35 | 0.81 |
| Sex, male (%) | 20 (66.7%) | 18 (60.0%) | 0.79 |
| SOFA score | 7.23±2.60 |  |  |
| Sepsis due to | | | |
| Peritonitis | 13 (43.3%) |  |  |
| Pneumonia | 2 (6.7%) |  |  |
| Multiple injuries | 15 (50%) |  |  |

Supplemental table 2
