# Supplementary figures and images for: Correction to: Cytochrome P450 1A1 enhances inflammatory responses and impedes phagocytosis of bacteria in macrophages during sepsis
Source: Cell Commun Signal. 2020 May 18;18:74. doi: 10.1186/s12964-020-00597-8 (PMC7236199; doi:10.1186/s12964-020-00597-8)

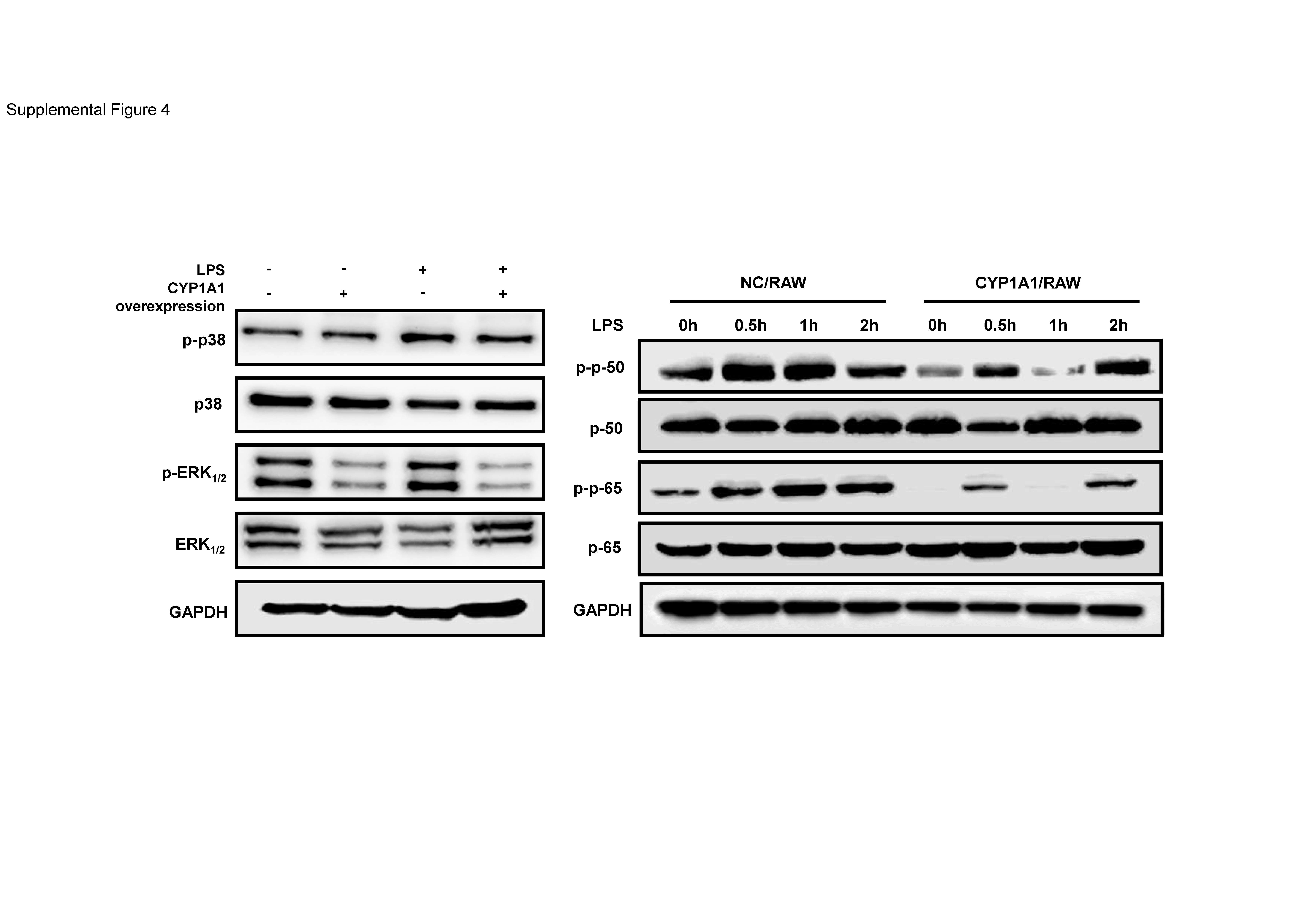

Supplement: Supplementary file 1 — Additional file 1: Figure S4. Validation of the NF-κB signalling pathway and different MAPK signalling pathways in LPS-stimulated CYP1A1/RAW and NC/RAW. [file 12964_2020_597_MOESM1_ESM.tif]
